# Modeling of the OX_1_R–orexin-A complex suggests two alternative binding modes

**DOI:** 10.1186/s12900-015-0036-2

**Published:** 2015-05-09

**Authors:** Lasse Karhu, Ainoleena Turku, Henri Xhaard

**Affiliations:** Division of Pharmaceutical Chemistry and Technology, Faculty of Pharmacy, University of Helsinki, P.O. Box 56, 00014, Helsinki, Finland

**Keywords:** Orexin-A, OX_1_ receptor, Peptide docking, G protein-coupled receptor, Pose selection, Multidimensional scaling, GPCR

## Abstract

**Background:**

Interactions between the orexin peptides and their cognate OX_1_ and OX_2_ receptors remain poorly characterized. Site-directed mutagenesis studies on orexin peptides and receptors have indicated amino acids important for ligand binding and receptor activation. However, a better understanding of specific pairwise interactions would benefit small molecule discovery.

**Results:**

We constructed a set of three-dimensional models of the orexin 1 receptor based on the 3D-structures of the orexin 2 receptor (released while this manuscript was under review), neurotensin receptor 1 and chemokine receptor CXCR4, conducted an exhaustive docking of orexin-A_16–33_ peptide fragment with ZDOCK and RDOCK, and analyzed a total of 4301 complexes through multidimensional scaling and clustering. The best docking poses reveal two alternative binding modes, where the C-terminus of the peptide lies deep in the binding pocket, on average about 5–6 Å above Tyr^6.48^ and close to Gln^3.32^. The binding modes differ in the about 100° rotation of the peptide; the peptide His26 faces either the receptor’s fifth transmembrane helix or the seventh helix. Both binding modes are well in line with previous mutation studies and partake in hydrogen bonding similar to suvorexant.

**Conclusions:**

We present two binding modes for orexin-A into orexin 1 receptor, which help rationalize previous results from site-directed mutagenesis studies. The binding modes should serve small molecule discovery, and offer insights into the mechanism of receptor activation.

**Electronic supplementary material:**

The online version of this article (doi:10.1186/s12900-015-0036-2) contains supplementary material, which is available to authorized users.

## Background

**The orexinergic system** is composed of two receptor subtypes, named orexin 1 and 2 receptors (OX_1_R and OX_2_R respectively), and of two agonistic peptide ligands, orexin-A and orexin-B [1]. Orexin receptors are mainly found in the central nervous system, but also in the periphery (gastrointestinal track, pancreas, adrenal gland and adipose tissue) [2]. Certain cancer cell lines also express OX_1_ receptors, whose activation induces apoptosis [3]. The endogenous orexin peptides induce feeding and wakefulness, and malfunctions of the orexin system are one of the reasons behind narcolepsy in mice, dogs and humans [2]. Small molecules (i.e. not peptides) have been developed to act as orexin receptor antagonists [4]. As expected, antagonists have opposing effects to orexin peptides; reduced feeding [5] and induction of sleep [4]. The first drug targeting the orexin receptors, the antagonist suvorexant (Belsomra®), has recently reached the market in the United States of America and in Japan.

Orexin peptides and receptors were discovered independently in 1998 by two research groups. Sakurai and co-workers discovered two peptides that produced robust Ca^2+^ elevation through activation of two receptors which they expressed in CHO cells [1]. The two peptides were named orexin-A and -B, and the receptor subtypes were designated as OX_1_ and OX_2_ receptors according to the Greek word for appetite, *oreksis* (ὄρεξις), since the peptides induced feeding in mice. De Lecea and co-workers discovered about simultaneously a mRNA sequence expressed in hypothalamus that encodes the precursor of the two peptides [6]. They named the peptides hypocretins 1 and 2.

**The orexin peptides** are produced as a 131-amino acid (in human) precursor, prepro-orexin, which is enzymatically cleaved to produce one unit of each peptide [1]. Human orexin-A is a 33-amino acid peptide containing two intramolecular disulfide bridges (Cys6–Cys12, Cys7–Cys14), an N-terminal pyroglutamoyl residue, and an amidated C-terminus [1]. Human orexin-B is composed of 28 residues and is amidated on its C-terminus like orexin-A, but lacks the disulfide bridges [1]. While the N-termini of the peptides are different, the C-termini are near-identical (11 out of 15 amino acids are identical). The receptor-bound conformations are not known, but NMR-structures for both peptides in buffered water solution have been solved [7,8]. Orexin-B comprises two helical parts (helix I: Leu7–Gly19 and helix II: Ala23–Met28) joined with a short linker or hinge (Asn20–Ala22) [7], whereas orexin-A has three helical sections (helix I: Leu16–Ala23, helix II: Asn25–Thr32 and helix III: Cys6–Gln9) [8]. The peptides were observed in multiple conformations: orexin-A is either in bent or straight conformation across the set of 30 NMR models [8], while the single model derived for orexin-B shows the hinge bent opposite to the conformation of orexin-A [7] (Additional file 1).

Mutations on the orexin peptides have shown that the C-terminal residues and the amidation of the C-terminus are the most important factors for receptor activation [9-12]. The N-terminus is not as important, as both peptides retained activity when truncated down to a C-terminal fragment of 19 residues [9-11]. Further truncation lowered the maximal response, but fragments as short as 12 amino acids still retained some activity [9-11]. No key residues have been found in the N-terminal part of the peptide.

**The orexin receptors** OX_1_R and OX_2_R are G protein-coupled receptors (GPCRs) that in human are composed of 425 amino acids (OX_1_R) and 444 amino acids (OX_2_R) [1]. As GPCRs, the overall structure of orexin receptors consists of seven helical transmembrane segments (TM1–7) connected by three intra- and three extracellular loops (ICL1–3 and ECL1–3 respectively), an extracellular N-terminus and an intracellular C-terminus, confirmed by the recent crystal structure of OX_2_R [PDB:4S0V] [13] that was solved while this manuscript was under review. The OX_2_ receptor has the conserved disulfide bridge connecting TM3 and ECL2, as was expected based on the receptor sequences. Most likely the OX_1_R will also have this bridge formed by Cys119^3.25^ and Cys202^xl2.50^.^a^ Both receptors have also suitable cysteines for C-terminal palmitoylation (Cys375 and Cys376 in OX_1_R), which is observed in most crystallized GPCRs. The human OX_1_R and OX_2_R share a full-length pairwise sequence identity of 64%, and without terminals and ICL3, the sequence identity of the TM bundle is up to 80%. Orexin-A is equipotent towards both receptor subtypes, whereas orexin-B is equipotent with orexin-A towards OX_2_R but 10-fold less potent in activating OX_1_R [10,11].

**Figure 1:**
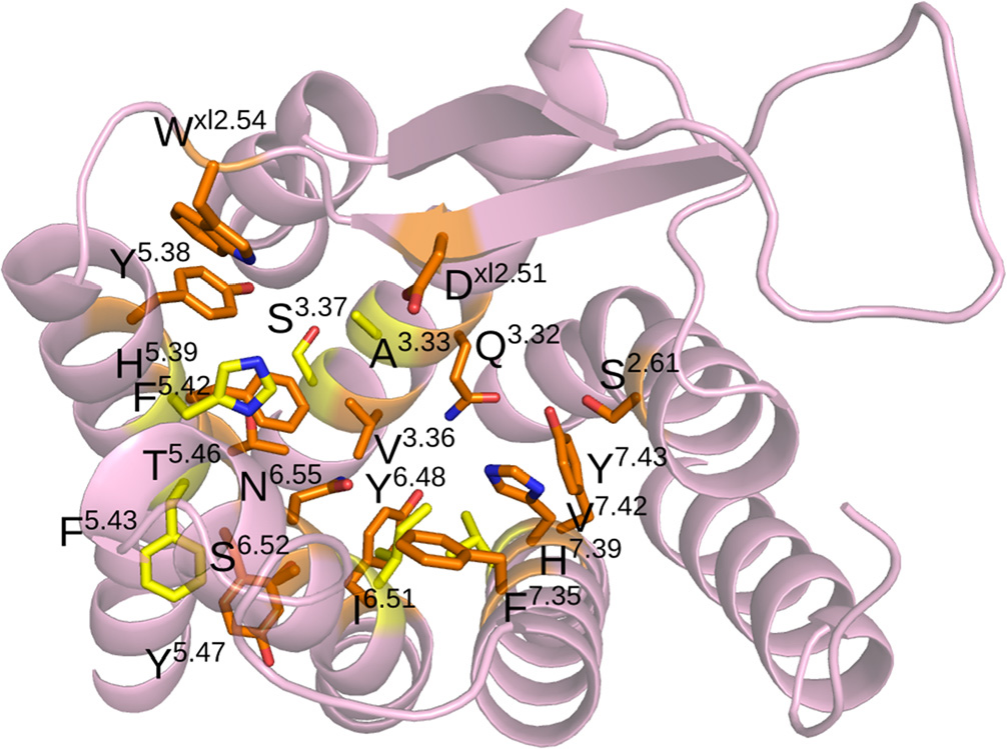
Point-mutated residues on the orexin receptors. Orange: mutation impaired the orexin-induced receptor activation in one or both subtypes; yellow: mutation did not alter the receptor function significantly [14,15].

The receptors have been mutated [14,15] and chimeras of OX_1_R and OX_2_R have been constructed [15,16] to study the contributions of different amino acids to interactions with ligands (Figure 1). Alanine mutations of OX_1_R residues Gln126^3.32^, Val130^3.36^, Asp203^xl2.51^, Trp206^xl2.54^, Tyr215^5.38^, Phe219^5.42^, Tyr224^5.47^, Tyr311^6.48^, His344^7.39^, and Tyr348^7.43^ decreased the potency and/or maximum response of orexin-A [14]. A similar study conducted on OX_2_R discovered that mutations of Thr231^5.46^ and Asn324^6.55^ (corresponding to Thr223 and Asn318 in OX_1_R) to alanine led to a 10-fold decrease in orexin-A potency [15]. This indicated that the orexin receptor ligand binding pocket is lined by residues from TMs 3, 5, 6 and 7 as well as ECL2, which was confirmed by the crystal structure of OX_2_R bound to suvorexant [13].

**Computational modeling** is a powerful tool to gain insight in the binding of the orexin peptides and the interactions leading to receptor activation. The prospective GPCR Dock studies [17-19] have shown that the transmembrane region of GPCRs can be reliably modeled and that computational tools are getting better at recreating receptor–ligand complexes. However, peptide docking without a known bioactive conformation remains challenging in part due to the inherent flexibility of peptides. In GPCR Dock 2010, the task of modeling chemokine receptor CXCR4 in complex with a synthetic 16-residue cyclic peptide proved difficult, since available templates had only distant homology to CXCR4 and the binding interactions were poorly characterized [18]. Recently, peptide docking software such as HADDOCK [20] (originally designed for protein–protein docking), Rosetta FlexPepDock [21], and DynaDock [22] have been developed. These software were tested with peptides ranging from 2 to 16 residues, often binding into a shallow groove on the protein surface [20-22]. Buried binding sites and helical peptides have been problematic [20,21]. Concerning GPCRs, peptides are docked with multiple methods; a rigid docking can be followed by a short molecular dynamics simulation [23-26], or semi-flexible methods can be used, such as Glide with induced fit [27] or GOLD, which allows rotamer-library-based side-chain rotation for selected residues [28]. Genetic algorithms can be used to produce changes to peptide backbone conformation [29]. In this study, we have used ZDOCK in combination with RDOCK to perform an exhaustive mapping of the OX_1_R binding site while allowing limited peptide and receptor flexibility. ZDOCK and RDOCK were originally developed for protein–protein docking and refinement [30,31], but they are also usable in peptide docking, which became evident in the GPCR Dock 2010 assessment, where one of the best peptide-docking results came from a group utilizing ZDOCK [18].

Previously, Heifetz and co-workers have aimed to establish a binding mode for orexin peptides to orexin receptors [28]. In their study, the dopamine D3 receptor served as a template for orexin receptor modeling. To account for protein flexibility, receptor conformations for docking were harvested from a short molecular dynamics simulation, and certain side chains in both receptor and ligand were allowed to adopt different rotamers. However, recent crystal structures for peptide-binding GPCRs have shown features such as the β-hairpin in the ECL2 that their models lack, and thus their results need to be updated.

Here, combining the data from the mutational studies conducted on orexinergic system and the crystal structures of peptide-binding GPCRs neurotensin receptor 1 (NTSR1), chemokine receptor CXCR4, and the recent crystal structure of the OX_2_R, we have constructed 3D-models of the OX_1_R. An exhaustive docking algorithm allowed mapping of the available space for orexin-A within the receptor cavity. Based on the molecular interactions observed in the docking results, we propose two alternative binding modes for orexin-A into OX_1_R. Studying these binding interactions will increase the understanding on the mechanisms by which the orexin peptides activate their cognate receptors, and provide a general framework to understand peptide-binding GPCRs.

## Methods

### Structural alignment of GPCRs

**Figure 2:**
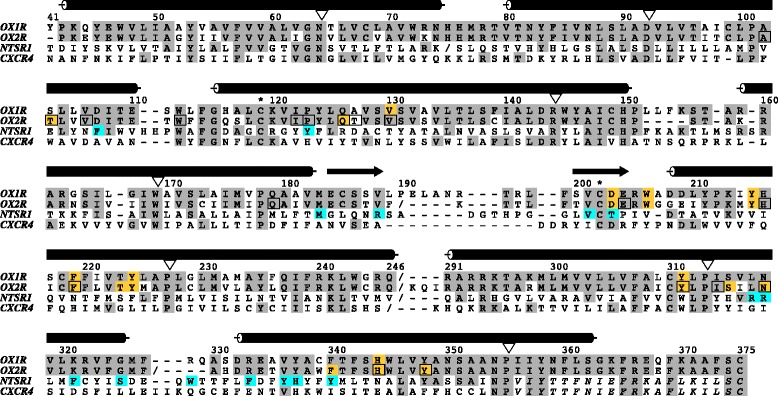
Sequence alignment used in homology modeling. Target sequence: OX_1_R [UniProt:O43613]. Template sequences: OX_2_R [PDB:4S0V], NTSR1 [PDB:4GRV] and CXCR4 [PDB:3ODU]. Orange: orexin receptor residues found to be important by site-directed mutagenesis. Cyan: NTSR1 residues that interact with neurotensin_8-13_. Boxed: OX_2_R residues within 4 Å of suvorexant. Italics: helix 8 from dopamine D3 receptor [PDB:3PBL]. Cylinders and arrows: TM helices and β strands seen in template structures. Numbering refers to OX_1_R. Triangle: x.50 residue. *: TM3–ECL2 disulfide bridge. Gray: identical residues between OX_1_R and templates. Illustrated with Alscript [57].

In order to identify structurally conserved regions, we superposed a total of 19 GPCR crystal structures available on RCSB Protein Data Bank (PDB) with Discovery Studio 3.5 [32]. Lysozyme chains were removed. A sequence alignment was derived from the superposition (Additional files 2 and 3). OX_1_R sequence was added manually to the alignment based on conserved motifs on each transmembrane helix [33]. We initially based the orexin receptor sequence alignment in the ECL2-region on the observation that all available crystallized peptide binding GPCRs — chemokine receptor CXCR4, neurotensin receptor 1 (NTSR1) and the four opioid receptors (mu, kappa, delta, and nociceptin) [34-39] — incorporate a similar β-hairpin fold of ECL2, composed of two five-residue β strands (in OX_1_R residues 184–188 and 200–204, see arrows in Figure 2 and in Additional file 2) and a turn of variable length (4–10 residues) between them. For OX_2_R, this hairpin structure was confirmed by the crystal structure, although not all amino acids in the turn were resolved [13]. In the crystal structures, the first β strand follows directly the TM4 and the second ranges from xl2.48 to xl2.52. The conserved disulfide bridge between TM3 and ECL2 constrains the second β strand, and therefore the β hairpin, above TM3.

### Template selection for homology modeling

Based on the structural alignment, the phylogenetic analysis of GPCRs [40], and the shapes of the observed binding pockets, we initially selected the crystal structure of the neurotensin receptor 1 [PDB:4GRV] [35] as a primary template for homology modeling. At the time, NTSR1 was the only crystallized receptor from the β branch of rhodopsin-like GPCRs where orexin receptors are found [40]. Like orexin receptors, the NTSR1 is also naturally activated by a peptide ligand, neurotensin. Neurotensin_8–13_ fragment has been co-crystallized with the receptor, but there is no G protein (or an antibody mimicking it), and thus the receptor conformation is not fully that of an activated GPCR [35]. While this article was under review, the crystal structure of OX_2_R in complex with the antagonist suvorexant was released [13]. To incorporate these recent data into our study, we utilized also the OX_2_R crystal structure as a template for homology modeling.

The NTSR1 crystal structure entails a binding cavity constricted by the TM6; the extracellular end of the helix is tilted towards the binding cavity, narrowing the cavity and limiting the exposure of the TM5 residues to the binding cavity. Therefore, we built two secondary homology models with more open binding cavities. One secondary model was based on the chemokine receptor CXCR4 [PDB:3ODU] [34], which naturally binds a small protein, although the receptor was crystallized with synthetic ligands. The CXCR4 crystal structure shows a more open binding cavity than the NTSR1. For the other secondary model, we constructed a modified NTSR1 structure template (NTSR1_TM6) by rotating the TM6 in the NTSR1 to occupy the same space as TM6 in CXCR4; this was done with Maestro 9.3.5 [41].

Neither selected crystal structure shows the 8th helix parallel to membrane plane observed in many other GPCR crystals. We selected dopamine D3 receptor [PDB:3PBL] [42] as a template for the 8th helix. Residues after Pro^7.50^ in NTSR1, CXCR4 and NTSR1_TM6 were replaced by those of dopamine D3 after careful superposition of TMs 1 and 7 of crystal structures. This was a cosmetic step that most likely does not affect the docking results. In retrospect, a more recent X-ray crystal structure of the NTSR1 [PDB:4BUO] [43] shows an intracellular assembly with the canonical TM8, as also does the recent crystal structure of the OX_2_R [13].

### Model building

Models of OX_1_R consisting of the residues Tyr41^1.27^–Gln246^5.69^ and Arg291^6.28^–Cys375 were built using the four templates mentioned above. The N- and C-termini, and ICL3 were omitted as there were no suitable templates. Homology modeling was carried out with MODELLER 9v8 [44], a comparative protein modeling program, using default settings. Pairwise alignment of OX_1_R with the templates was fine-tuned in tandem with model building (Figure 2). Ten models were derived from each template structure.

### Model evaluation

The 40 models were evaluated visually to eliminate unreasonable constructs and to select four models for docking, each displaying an open binding cavity and resulting from a different template. Modeller DOPE scores did not differ significantly between models of same origin. We selected the models based on the conformations of ECL2 and ECL3. The ECL2, especially the turn between the strands of the β hairpin, was required to show a secondary structure similar, and occupying roughly the same space, as those observed in the crystal structures of peptide binding receptors. The ECL3 was required not to constrict the entrance of the binding cavity.

### Orexin peptide conformation for docking

We used the straight α-helical conformation of orexin-A (the second NMR model in [PDB:1WSO] [8]) in this study, as the bent conformation did not fit the predicted binding site in a preliminary docking. Instead of the full orexin-A peptide, a fragment comprising of residues 16–33 was used. This fragment retains biological activity [10], and using it avoids the problem of the N-terminus of the intact orexin-A colliding with the extracellular loops of receptor models and limiting the conformational space.

### Docking of orexin peptides with ZDOCK and RDOCK

Prior to docking, the receptor models and the peptide fragment were converted to CHARMm atom types as required by the docking program. ZDOCK [30], an exhaustive initial-stage docking algorithm for protein–protein complexes, was used with default settings. We filtered the docking poses by accepting only the poses where the ligand C-terminal residues (shown to be crucial for activity, see Background) were part of the receptor–ligand interface and the ligand did not traverse between the TM helices into the space occupied by the cell membrane. The poses were refined with RDOCK [31] using default settings. RDOCK is a CHARMm force field based refinement algorithm that performs limited molecular dynamics to fine-tune receptor–ligand complexes from ZDOCK. RDOCK uses a two-stage scoring function; van der Waals energy is first calculated to discard docking poses with clashes and then the poses are scored based on desolvation and electrostatic energies. Accelrys Discovery Studio 3.5 [32] was used as an interface to ZDOCK and RDOCK and to visualize the results.

### Data analysis on the docking poses

We clustered the refined docking poses modelwise using an algorithm devised by Daura and co-workers [45], implemented in MATLAB [46]. In short, a matrix of all pairwise root mean square deviations (RMSD) of the peptide α carbons is calculated. The pose with most neighbors (here RMSD < 3 Å) is flagged as the cluster seed, and the neighbors are included in the cluster and removed from the pool of poses. The process is repeated until no two poses are closer than the cutoff. For cluster scoring, we used the median RDOCK score of the poses in each cluster.

For multidimensional scaling, we pooled all docking poses across models and calculated all pairwise RMSD values. MATLAB was used to reduce dimensions to two (*mdscale* function) and to visualize the outcome. Solvent accessible surface area for the peptide ligand was calculated with Naccess [47] (default settings). For measurements of ligand depth, the *z*-coordinate (*z*-axis normal to the membrane plane) of the Leu33 α carbon (Cα) was used. The zero-plane was set to the Cα's of Thr223^5.46^, Tyr311^6.48^ and Tyr348^7.43^.

We assessed the rotation of the peptide ligand around its helical axis by drawing a vector towards the side chain of His26 (from Ala28 Cα to His26 Cα) in *xy*-plane (the plane parallel to the membrane). By using a common initial point for the vectors, preferences in ligand orientation could be seen.

The contact mapping was carried out with MATLAB by calculating the pairwise distances between ligand atoms and atoms in the receptor residues in the binding cavity. Any pairwise distance between atoms closer than 4 Å was considered a contact. No differentiation between side-chain and main-chain atoms was done at this point.

## Results and discussion

### Homology modeling

The crystal structures of class A (rhodopsin-like) GPCRs show clear conservation within TM segments and short loops, as illustrated by the structural alignment. The sequence alignment within the TM region is unambiguous (Additional file 2). The ECL2 and ECL3 vary both in length and in conformation between receptors, but closely related receptors often show similarities; for example all peptide binding receptors (NTSR1, CXCR4, and the four opioid receptors mu, kappa, delta, and nociceptin, and also the recent OX_2_R structure [13,34-39]) show similar β-hairpin structures in the ECL2, although the segment between the β strands varies in length: three amino acids in CXCR4, nociceptin, delta, and mu opioid receptors, five in kappa opioid receptor, and nine in NTSR1 (see Additional file 2). The orexin receptors have a segment of 11 amino acids between the β strands, but five of them were not solved in the recent OX_2_R crystal structure. Also ECL3 differs in length among the OX_1_R and the crystallized receptors.

Orexin receptors have most class A GPCR-specific motifs; instead of the conserved Tyr3.51 (in the “DRY” motif) and Trp6.48 (at the bottom of the binding cavity, the “CWxP” motif), orexin receptors have Trp145^3.51^ and Tyr311^6.48^. As both substitutes are aromatic residues, the structural functions are likely to be conserved. In the extracellular half of the orexin receptor TM3, Pro123^3.29^ is present. This feature is common in the β branch of rhodopsin-like GPCRs, and a comparison retrospective to this work between the OX_2_R structure and the other crystallized class A GPCRs shows that the conformation of the TM3 remains unaltered by the proline.

Templates originally used in this study (NTSR1 and CXCR4) both have sequence identities of 23.6% (70 identical residues out of alignment length of 296) to human OX_1_R transmembrane bundle (Tyr41^1.27^–Gln246^5.69^ and Arg291^6.28^–Cys375). This level of sequence identity is usually considered poor for homology modeling, but the overall fold shared by the crystallized class A GPCRs was likely to be conserved also in the orexin receptors, which was confirmed by the OX_2_R crystal structure [13]. Within the transmembrane bundle, NTSR1 and CXCR4 both have six alignment gaps in comparison to OX_1_R. For NTSR1, all gaps fall into loops (Figure 2). In contrast, CXCR4, together with opioid receptors, shows a gap at 2.57 (2x551 according to the structure-based residue numbering proposed by the GPCRDB [48]), which results in the absence of a bulge shown by other crystallized class A GPCRs. CXCR4 has also a bulge-inducing insertion at 4.47 (4x471) (Additional file 2), while other alignment gaps occur in the loops. These are present in our CXCR4–OX_1_R sequence alignment used for homology modeling (Figure 2). As the TMs 2 and 4 are only marginally exposed to the interhelical cavity, the effect of possible misalignment on the binding site of the CXCR4-based model is negligible.

Considering the conserved TM3–ECL2 disulfide bridge, human orexin receptors have two cysteine residues in the ECL2: Cys185/193 and Cys202/210 (OX_1_R/OX_2_R). Based on the sequence alignment, and the fact that rat OX_2_R has arginine instead of Cys193 [UniProt:P56719], we assumed that the Cys202/210 would be involved in the disulfide bridge with Cys119/127^3.25^. The crystal structure of OX_2_R indeed shows the disulfide bridge between Cys127 and Cys210 (corresponding to Cys119 and Cys202 in OX_1_R).

### Homology models

**Figure 3:**
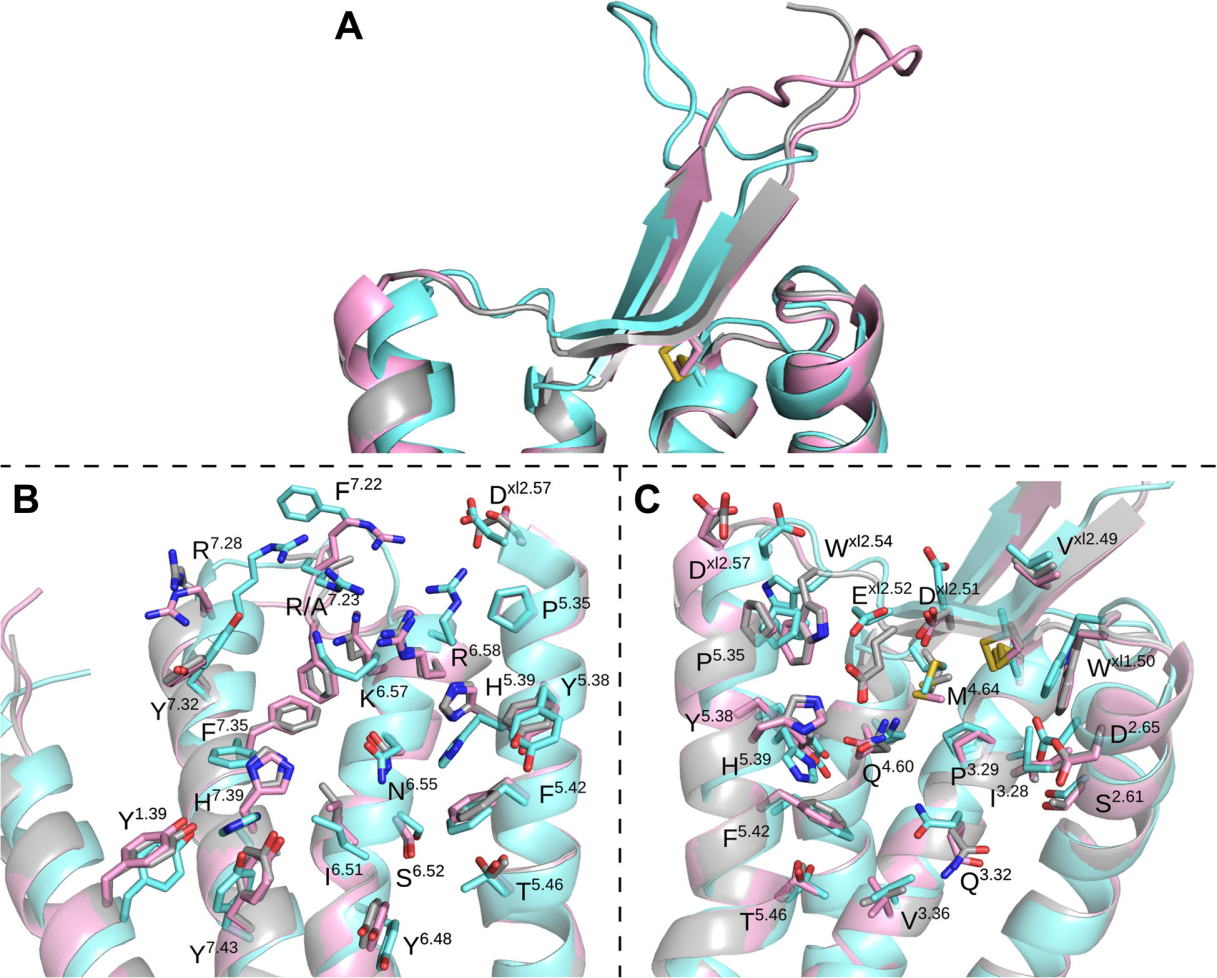
Comparison of the homology models. Pink and cyan: OX_1_R homology models based on OX_2_R and NTSR1 respectively. Gray: OX_2_R crystal structure [PDB:4S0V]. **(A)** Conformation of ECL2. **(B** and **C)** Residues facing the receptor cavity from TMs 1, 5–7 and TMs 2–5 respectively.

Originally three models were selected, one from each template, among the 30 generated models. Later a fourth model, based on the recent OX_2_R structure, was included from a set of ten constructed models (the models are available as Additional file 4). Overall, the main chains superimpose well among the models, and in retrospect also to OX_2_R crystal structure, but some differences arise especially in the loops and in the TM6 (Figure 3, Additional file 5). The side chain conformations show more variance, but the difference in the backbone conformation is more significant to the docking, as the applied docking protocol is capable of adjusting the side chains but not the protein backbone.

Our original primary model, based on NTSR1, has a narrow cavity due to the inward tilt of the TM6 (volume of ca. 1400 Å^3^, calculated with 3V-web server [49]). In retrospect, the overall shape and size of the cavity in the NTSR1-based model closely resembles that of the OX_2_R-based model, which in turn is near-identical in conformation to the OX_2_R crystal structure (pairwise heavy atom RMSD 1.07 Å). The ECL2 of the NTSR1-based model adopts a β-hairpin structure similar to the OX_2_R-based model, but the turn between the strands varies in conformation due to Modeller loop modeling (Figure 3A). The transmembrane bundle of the NTSR1-based model superimposes well to the OX_2_R-based model, although the side-chain rotamers vary (Figure 3B,C). The heavy atom RMSD between NTSR1- and OX_2_R-based models for binding-site-facing residues is 3.4 Å.

Location of the TM6 is a major difference between the OX_2_R-based model and the NTSR1_TM6- and CXCR4-based models (Additional file 5). Due to the outward-leaning TM6, the binding cavities of these two models are more open and spacious (ca. 2000 Å^3^). Also these models show the β-hairpin structure in the ECL2 (Additional file 5), and the TMs 2–5 superimpose well to the OX_2_R-based model, again with varying side-chain rotamers. The different conformation of the TM6 in the NTSR1_TM6- and CXCR4-based models leads to poor superimposition of binding site residues over the OX_2_R-based model. The TM7 is similarly located in all models, but in the CXCR4-based model the TM7 shows a counterclockwise rotation of ~50° around the helical axis in comparison to the other models, which slightly alters the set of residues that face the binding cavity. The heavy atom RMSD of binding-site-facing residues of the NTSR1_TM6- and CXCR4-based models in comparison to OX_2_R-based model is 4.0 Å and 4.7 Å respectively.

### Docking results

Docking into the OX_2_R-based model produced 1099 docking poses, and to the NTSR1-based model 1164 docking poses. Secondary models based on CXCR4 and NTSR1_TM6 produced 1180 and 858 poses respectively. The poses were clustered into 53, 50, 68 and 48 clusters based on pairwise RMSD.

**Figure 4:**
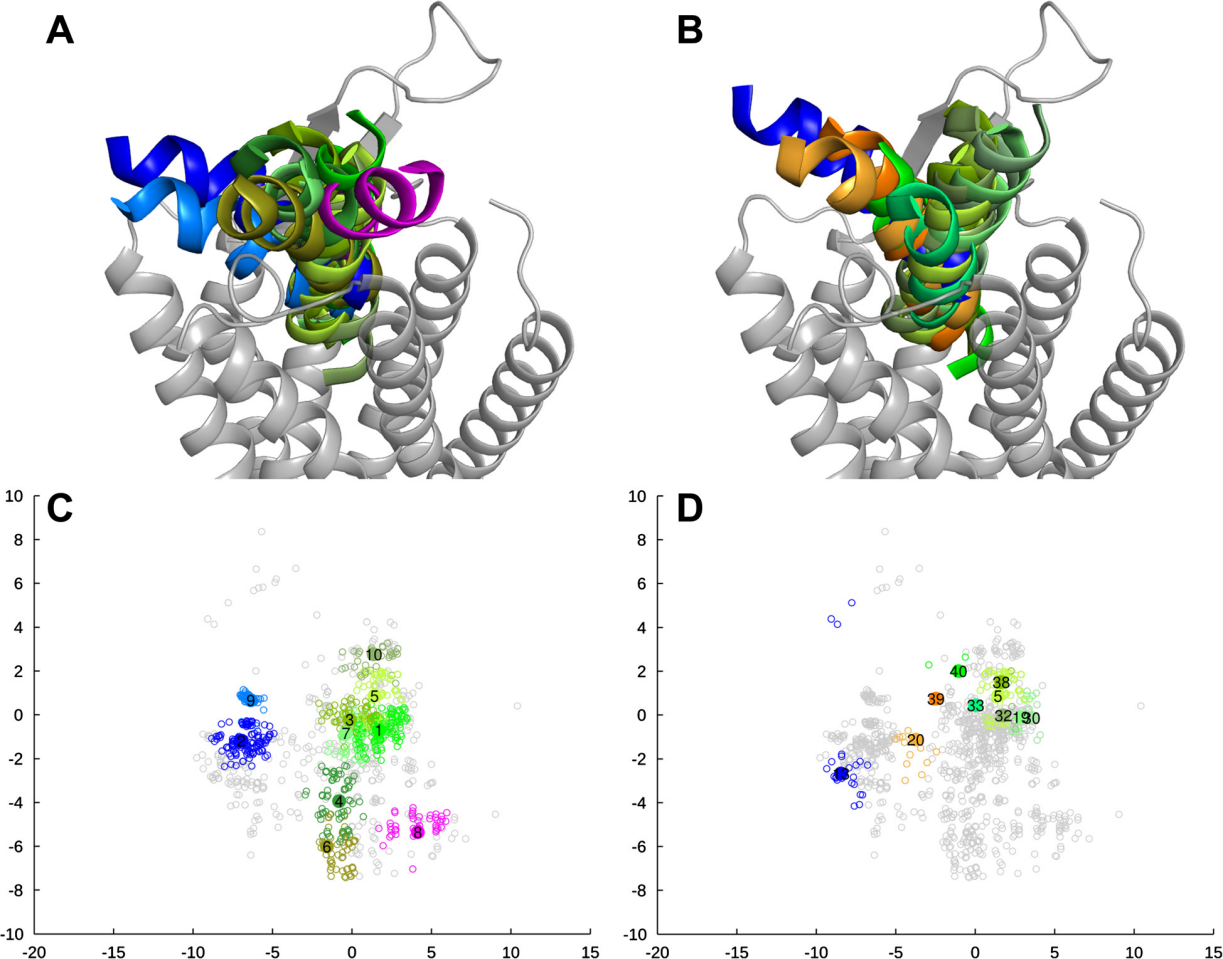
3D-representations for the docking pose clusters and scatter plots from multidimensional scaling, OX_2_R-based model. **(A, C)** Ten largest clusters; **(B, D)** Ten top-scoring clusters. In panels A–B, the TM1 is on the right. Multidimensional scaling shows the clusters (colored; numbers refer to size ranking) in respect to the pool of docking poses (gray). Poses leaning towards the TMs 1–2 are shown in shades of red/magenta, poses leaning to the TMs 5–6 are cyan, blue or purple, and poses vertically in the cavity are orange, green or dark green (See Additional file 6 for all clusters and the color division). The coloring is consistent between 3D-representations and plots.

**Figure 5:**
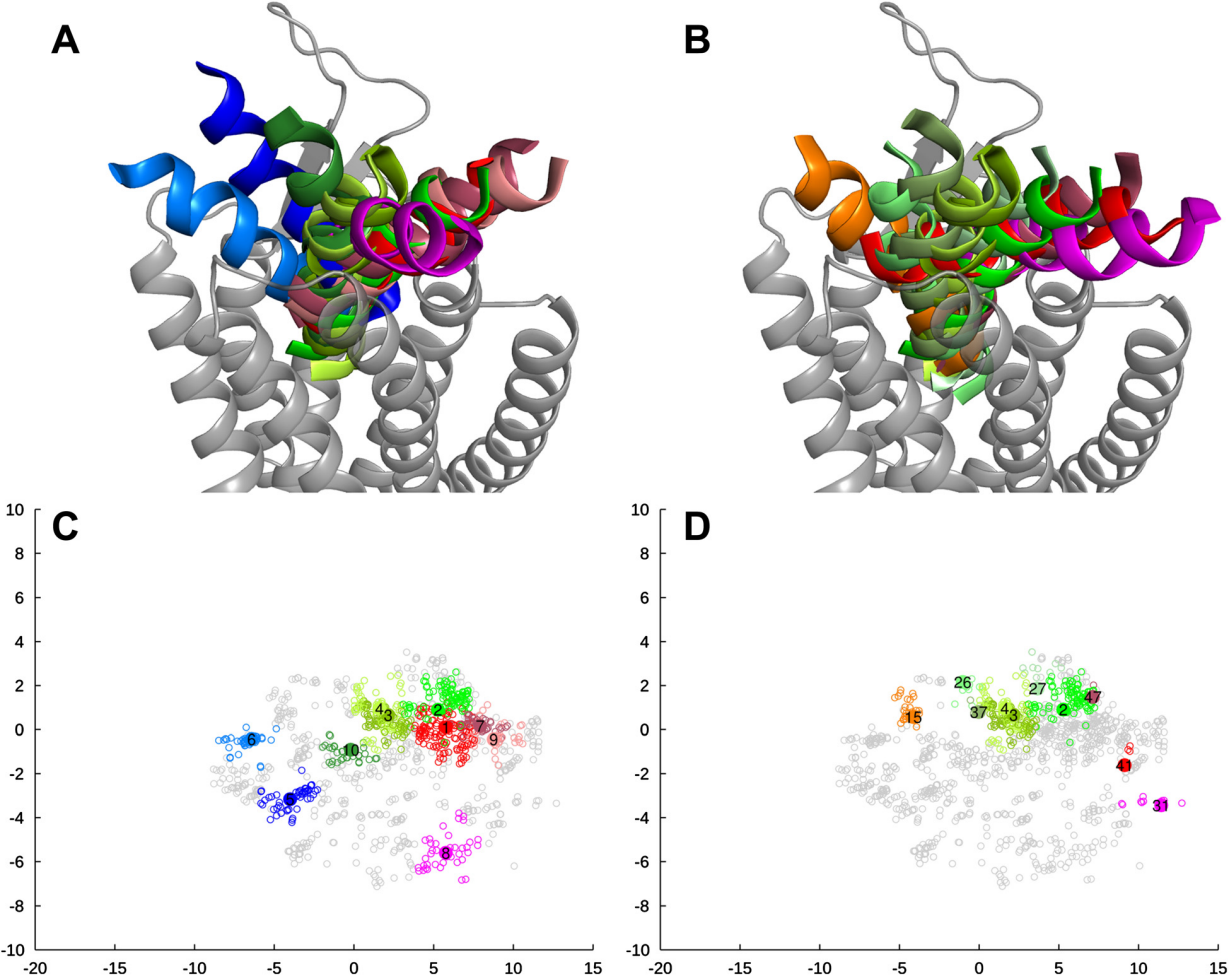
3D-representations for the docking pose clusters and scatter plots from multidimensional scaling, NTSR1-based model. **(A, C)** Ten largest clusters; **(B, D)** Ten top-scoring clusters. The view and color coding is as in Figure 4.

In the OX_2_R-based model, the docking poses form a tight “bouquet” (Figure 4A, Additional file 6), with some poses leaning over to the TM5-side of the cavity. Top-scoring clusters occupy a tight space vertically in the middle of the binding cavity, again with some clusters leaning over to TM5 (Figure 4B). In the NTSR1-based model, the available space for the peptide ligand is fan-shaped (Figure 5A, Additional file 6), which is a result of the narrow interhelical cavity. The top-scoring clusters tend to show a vertical ligand orientation with C-terminus deep in the cavity (Figure 5B). Few clusters show poses higher and slanted towards TM1.

The more spacious binding cavities of the secondary models result in wider distributions of docked poses. The CXCR4-based model has a more open binding site, which leads to a wide bouquet-like distribution of docking poses (Additional file 6). The same distribution is seen with the ten largest and the top-ten-scoring clusters. However, top-ranking clusters reveal no preferences in ligand position. For the NTSR1_TM6-based model, the docking poses fall into two groups; the poses residing at the TM5-side of the cavity, and the poses leaning towards the TM1-side of the cavity (Additional file 6). The ten largest clusters show similar distribution, but the TM5-side and an upright orientation is favored by the top-ten-scoring clusters.

Scores for the individual top-scoring poses varied modelwise. The NTSR1-based model produced the highest scores (the best score -17.86), and 35 docking poses had RDOCK score of -10 or less. The OX_2_R-based model produced 11 poses with RDOCK score below -10 (best score -14.47), whereas the NTSR1_TM6 and CXCR4-based secondary models have 8 and 6 poses with scores < -10, best scores being -12.15 and -11.83 respectively. The narrow binding cavities of the NTSR1- and OX_2_R-based models may enable the formation of more favorable interactions than the secondary models with more open binding cavities. The average docking pose scores show only minor differences (-1.50, -1.13 -0.68 and -1.05 for NTSR1-, OX_2_R-, NTSR1_TM6- and CXCR4-based models respectively).

RDOCK has originally been designed to refine and score protein–protein complexes, not docked peptide ligands. The scoring, however, relies on calculated desolvation and electrostatic energies, so it should also be applicable to peptide docking. In our study, the connection between the 3D-location of the docking pose and the score can be seen in the score differences among clusters, and in the distribution of top-ranked 5 and 10% of the docking poses into clusters (Additional file 7). One-way variance analysis (ANOVA) shows that the differences between the cluster scores are statistically significant (data not shown). The scoring shows no bias towards deep ligand binding, as it appears to be uncorrelated with both the solvent accessibility of the peptide ligand and the depth of binding (Additional file 7). Therefore it appears reasonable to focus further analysis on the top-ranking individual poses.

### Top-ranking poses

**Figure 6:**
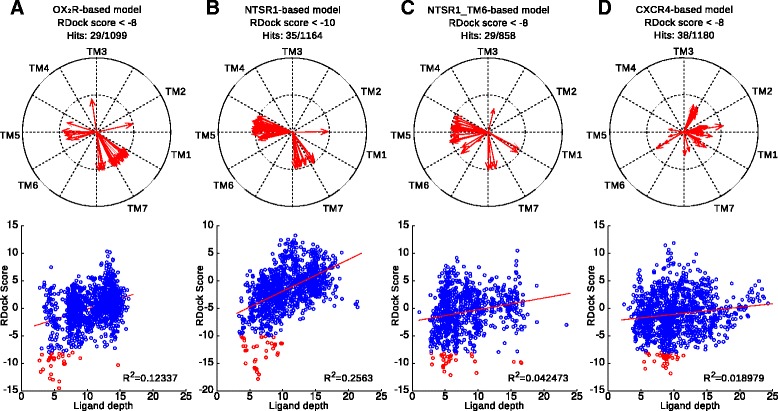
Modelwise depth and orientation of top-ranking docking poses. **(A)** OX_2_R-based model, **(B)** NTSR1-based model, **(C)** NTSR1_TM6-based model, **(D)** CXCR4-based model. Direction of His26 side chain plotted with arrows, as seen from the extracellular side. For vertical peptides the arrows touch the inner circle, for tilted peptides the arrows are shorter. Scatter plots show RDOCK score as a function of binding depth. Poses below the filter threshold are shown in red.

For each model, top-ranking poses were selected for closer examination. For the NTSR1-based model, the 35 docking poses that had RDOCK score of -10 or lower were used. As the top-scores for the other models were in general worse, a filter of RDOCK score < -8 was applied to yield 29, 38 and 29 docking poses from the OX_2_R-, CXCR4- and NTSR1_TM6-based models respectively. In all four models, the majority of top-ranking poses show the peptide ligand about vertically fairly deep in the binding cavity (Figure 6). The NTSR1-based model shows ligand depth of 3.7–9.9 Å (median 6.1 Å, zero-level at Tyr311^6.48^ Cα, see Methods), whereas the OX_2_R-based model favors deeper binding (median 5.0 Å, 2.8–14.7 Å). Regarding the secondary models, the best poses from the NTSR1_TM6-based model are more diverse, and depths range from 2.9 Å to 16.6 Å (median 5.5 Å). The best docking poses from the CXCR4-based model are a bit higher, 5.5–11.4 Å (median 8.8 Å).

The rotational orientation was assessed as the direction of bulky residues close to C-terminus (His26, Leu30, and Leu33) in respect to the receptor (Figure 6). In each model, definite preferences are seen, although these preferences are not the same for all models. In the top-ranking poses from the NTSR1-based model and the NTSR1_TM6-based secondary model, the bulky residues face the TM5-side of the cavity in 70% of the poses. For the remaining top-scoring poses, the bulky residues face TM7. The preferences in the NTSR1_TM6-based model are not as strict as in the NTSR1-based model. The OX_2_R-based model also shows these two groups of docking poses, but the preference is reversed; the majority of the top-ranking poses (69%) shows the bulky ligand residues facing the TM7, whereas the TM5-facing poses are a minority (24%). The docking poses in the CXCR4-based secondary model have a different preference, where the majority of the poses has the bulky residues on the TM1-side of the cavity, or facing towards TM2–3-side of the receptor. This difference in the preferred orientation is not surprising, given that the docking poses in CXCR4-based model are in average ~2 Å closer to extracellular surface and thus have access to different areas of the binding cavity. This is likely caused by the bulky residues of TM7, especially His344^7.39^, which in the CXCR4-based model face the cavity more prominently due to the 50° counterclockwise rotation of the TM7. These modelwise preferences in orientation are clearly mirrored in the mapping of contact frequencies between ligand and receptor residues (Additional file 8).

### Two alternative binding modes

The top-ranking docking poses from the OX_2_R- and NTSR1-based models were divided into two categories based on the peptide rotational state. The binding mode with TM5-facing bulky residues (“TM5-mode”) was adopted by 31 poses (7 + 24 poses from the OX_2_R- and NTSR1-based models respectively), whereas 30 poses (20 + 10) show the bulky residues towards the TM7 (“TM7-mode”). The OX_2_R-based model shows two outliers that do not fall into these two categories, while the NTSR1-based model shows one (Figure 6).

**Figure 7:**
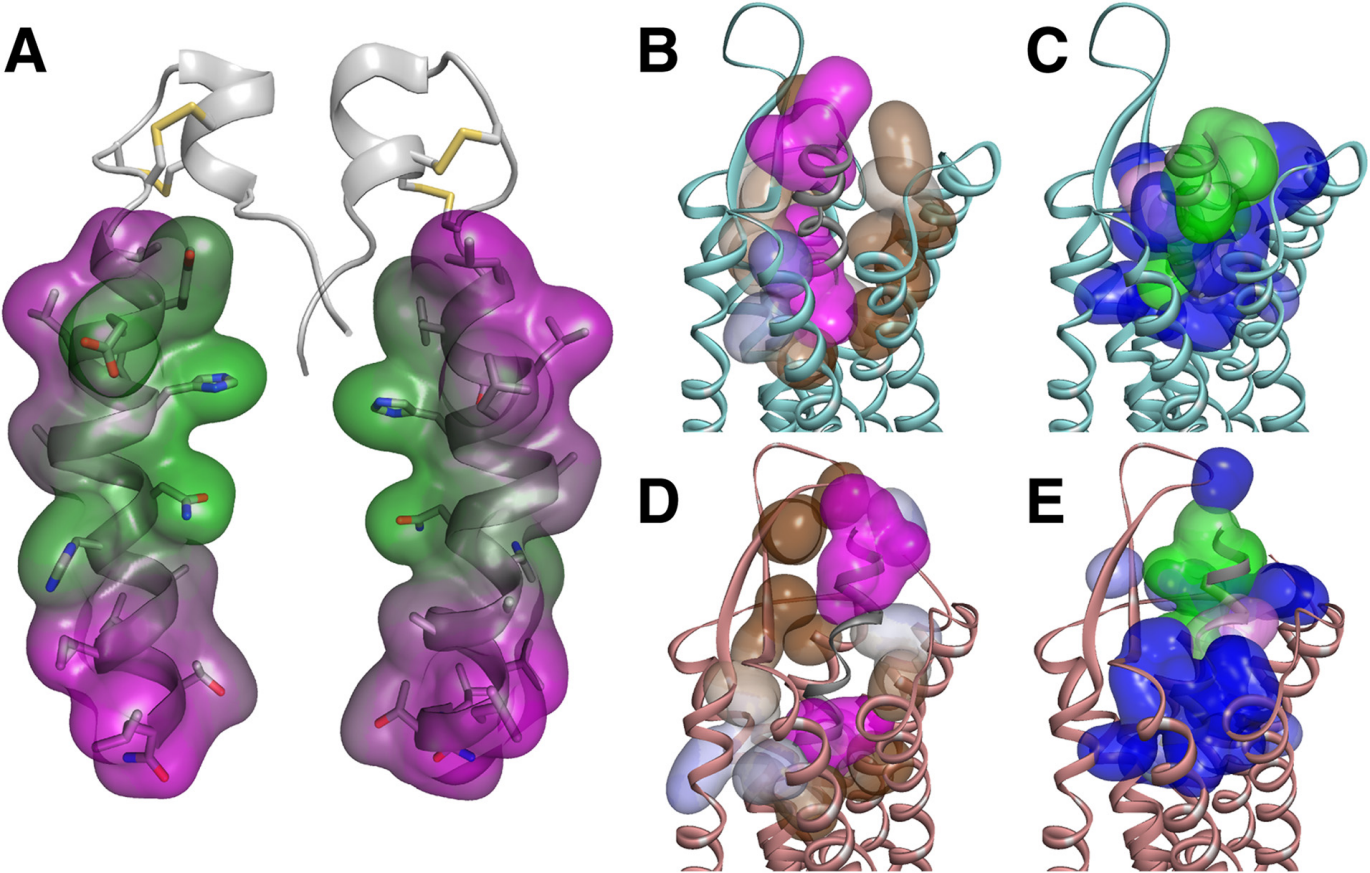
Orexin-A peptide and the surface complementarity of the two binding modes. **(A)** Orexin-A from opposite sides. **(B, C)** TM5-mode. **(D, E)** TM7-mode. Panels B and D show hydrophobic, and panels C and E hydrophilic surfaces. Receptor surfaces on color scale brown-blue (hydrophobic-hydrophilic), ligand surfaces magenta-green. The receptor surface has been drawn based on the side chain atoms of the residues that have atoms within 4 Å of the peptide ligand.

In both binding modes, the peptide C-terminus lies deep in the interhelical pocket, and forms reasonable interactions that take advantage of important amino acids (discussed in detail below). In the peptide N-terminus, the TM5-mode shows apparent better complementarity of hydrophobic and hydrophilic residues between the peptide and the receptor than the TM7-mode (Figure 7). Especially the hydrophobic amino acids in the peptide N-terminus (L16, L19, and L20) make a drastic difference between the binding modes. The TM5-mode shows these amino acids close to the ECL2 hairpin, partially shielded from the solvent (Figure 7B), whereas in the TM7-mode these amino acids are exposed to the solvent (Figure 7D). This exposure would remain the same with full orexin-A peptide as the disulfide-bridge-stabilized N-terminus lies on the opposite side of the peptide than the hydrophobic group of L16, L19 and L20 (Figure 7A). However, our models lack the receptor N-terminus, and both the conformation of the turn structure in the receptor ECL2 hairpin and the ligand N-terminus could be different. These factors could have extensive effect on the solvent exposure of hydrophobic ligand residues.

Both binding modes appear to be compatible with full-length orexin-A. The disulfide-bridge-stabilized N-terminus of orexin-A in the straight conformation would be close above ECL3 in the TM5-mode, whereas in the TM7-mode it would be near the hairpin-turn of the ECL2. In contrast, the bent conformation, which is more frequently seen in the solution NMR-studies, would not fit these binding modes, as the peptide N-terminus would clash into TM7 or receptor N-terminus in the case of the TM5-mode and into the ECL2 for the TM7-mode.

### Binding interactions

**Figure 8:**
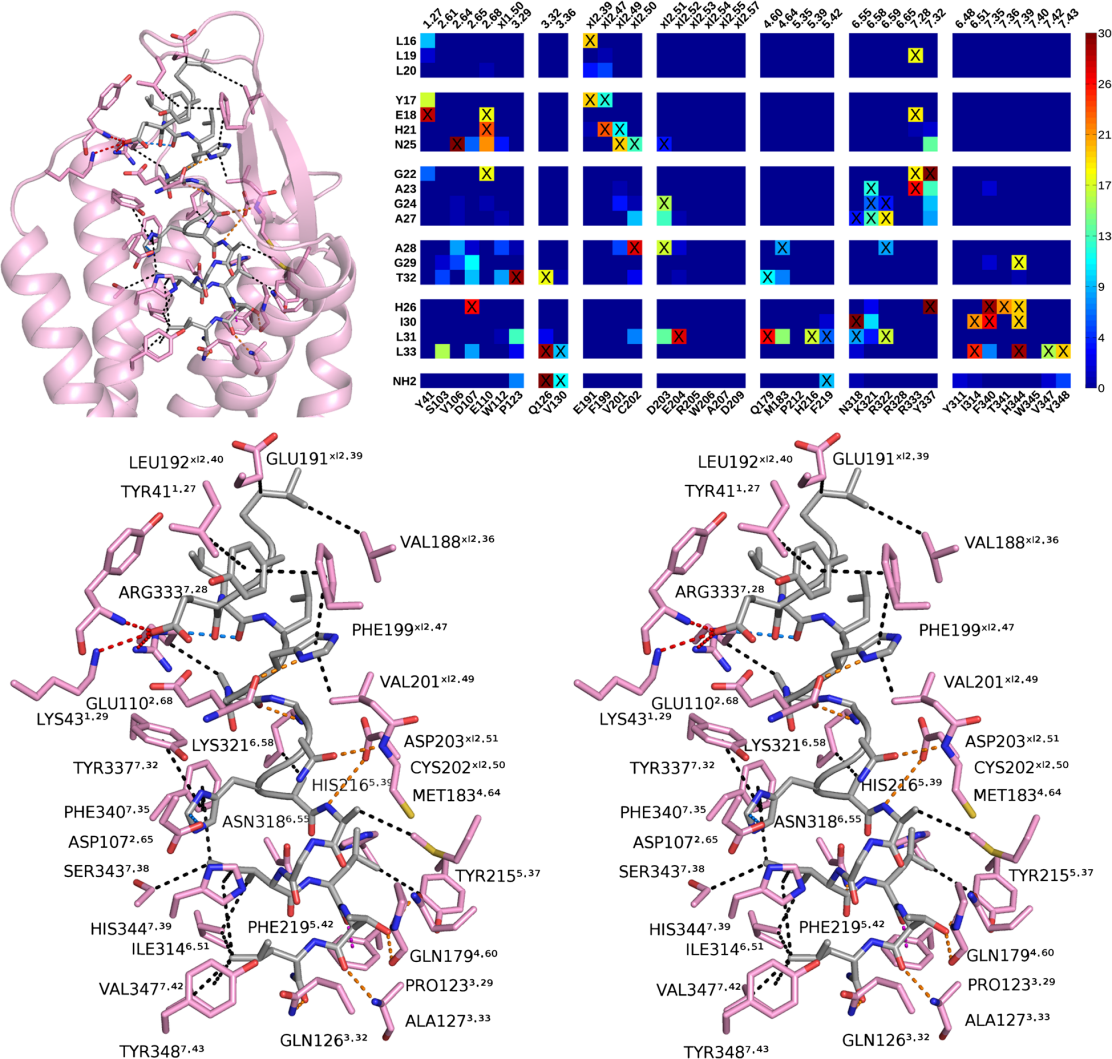
The TM7-binding mode. (Top left) Overview of receptor–ligand interactions. (Top right) Heatmap shows preferred peptide–receptor interactions (interatomic distance < 4 Å) within the high-scoring poses that adopt this binding mode. X: observed in the representative pose. (Bottom) A cross-eyed stereogram. Orange: hydrogen bond, red: salt bridge or charge-assisted hydrogen bond, blue: CH–O hydrogen bond, magenta: lone pair-π, black: hydrophobic.

**Figure 9:**
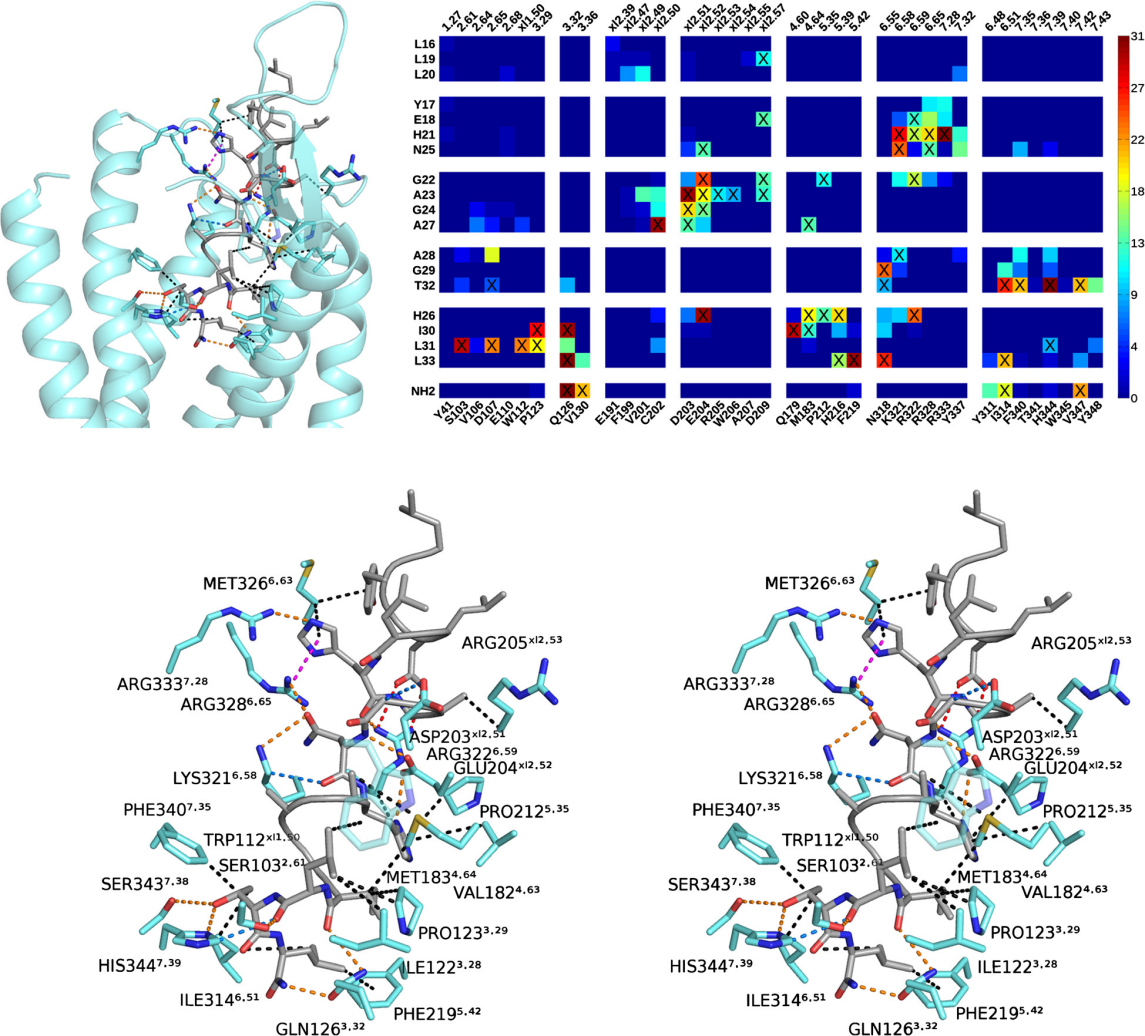
The TM5-binding mode. Overview of receptor–ligand interactions. (Top right) Heatmap shows preferred peptide–receptor interactions (interatomic distance < 4 Å) within the high-scoring poses that adopt this binding mode. X: observed in the representative pose. (Bottom) A cross-eyed stereogram. Color coding as in Figure 8, but magenta marks cation-π interaction.

For both binding modes, a representative pose was selected to illustrate binding interaction at the atomic level (Figures 8 and 9). The interactions are summarized in Table 1. In general, orexin-A presents two large hydrophobic surfaces, one close to each terminus. The polar side chains, the peptide backbone at the flexible hinge region and, at the last helical turn, the exposed carbonyls and the amidated C-terminus offer sites for hydrogen bonding and electrostatic interactions.

**Table 1:** **Binding interactions of the two presented binding modes**

**Ligand residue**	**Interactions with receptor residues**	
	**TM7-mode**	**TM5-mode**
Leu16	Alkyl - Val188^xl2.36^, Glu191^xl2.39^	-
Tyr17	Aromatic - Phe199^xl2.47^	Alkyl-π - Met326^6.63^
Glu18	Salt bridge - Arg333^7.28^, Lys43^1.29^	Salt bridge - Arg322^6.59^
	H-bond - Tyr41^1.27^ (backbone N)	
Leu19	CH–O to backbone - Arg333^7.28^	-
Leu20	-	-
His21	Aromatic - Phe199xl^2.47^	H-bond - Arg333^7.28^
	Alkyl-π - Val201^xl2.49^	Cation-π - Arg328^6.65^
	H-bond - Glu110^2.68^ (backbone carbonyl)	Alkyl-π - Met326^6.63^
		H-bond to backbone - Arg322^6.59^
Gly22	-	-
Ala23	Alkyl - Arg333^7.28^	Alkyl - Arg205^xl2.53^
	H-bond to backbone - Lys321^6.58^	
Gly24	-	CH–O hydrogen bond - **Asp203** ^**xl2.51**^
Asn25	H-bond - Cys202^xl2.50^ (backbone nitrogen)	H-bond - Arg328^6.65^, Lys321^6.58^ (putative)
		H-bond to backbone - Glu204^xl2.52^, Lys321^6.58^ (conventional or CH–O)
His26	Aromatic - Tyr337^7.32^, Phe340^7.35^	Alkyl-π - Arg322^6.59^, Val182^4.63^, Pro212^5.35^
	CH–O hydrogen bond - Asp107^2.65^	H-bond - Glu204^xl2.52^
		CH–O hydrogen bond - Pro212^5.35^ (carbonyl)
Ala27	Alkyl - Lys321^6.58^	Alkyl - Met183^4.64^
Ala28	Alkyl - Met183^4.64^	-
	H-bond to backbone - **Asp203** ^**xl2.51**^	
Gly29	-	H-bond to backbone - **Asn318** ^**6.55**^ (requires rotamer change)
Ile30	Alkyl-π - Phe340^7.35^, **His344** ^**7.39**^	Alkyl - Pro123^3.29^
	Alkyl - Ile314^6.51^, Ser323^7.38^	H-bond to backbone - **Gln126** ^**3.32**^
	H-bond to backbone - **Asn318** ^**6.55**^	
Leu31	Alkyl-π - **Tyr215** ^**5.37**^, His216^5.39^	Alkyl-π - Trp112^xl1.50^
	Lone pair-π from backbone - **Phe219** ^**5.42**^	Alky - Ile122^3.28^, Pro123^3.29^
		H-bond to backbone - Ser103^2.61^
		CH–O hydrogen bond to backbone - **His344** ^**7.39**^
Thr32	H-bond - Gln179^4.60^, Pro123^3.29^ (backbone carbonyl)	Alkyl-π - Phe340^7.35^, **His344** ^**7.39**^
		Alkyl - Ile314^6.51^
		H-bond - Ser343^7.38^, **His344** ^**7.39**^ (either to threonine hydroxyl or backbone carbonyl)
Leu33	Alkyl-π - **His344** ^**7.39**^, **Tyr348** ^**7.43**^	Alkyl-π - **Phe219** ^**5.42**^
	Alkyl - Ile314^6.51^, Val347^7.42^	Alkyl - Ile314^6.51^
	H-bond to backbone - **Gln126** ^**3.32**^	
NH2	H-bond - **Tyr311** ^**6.48**^ (requires rotamer change)	Close to **Gln126** ^**3.32**^ (unfavorable geometry for H-bond)

Interactions divided by type. Unless otherwise noted, the interacting atoms are side-chain atoms. “Requires rotamer change” denotes putative interactions which would take place if a receptor residue adopted a slightly different rotamer. Receptor residues whose mutation has been shown to be detrimental to orexin peptide binding are in bold.

**Figure 10:**
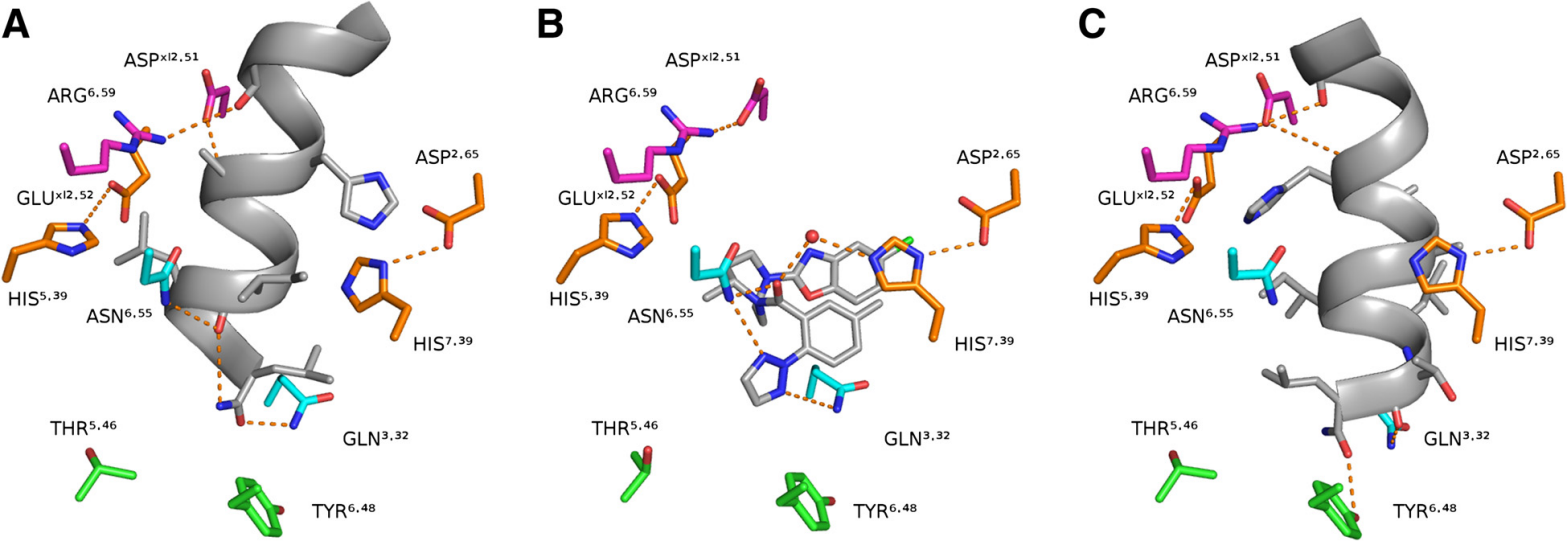
Comparison to suvorexant binding. **(A and C)** TM7- and TM5-modes respectively, **(B)** Suvorexant binding. Orange dash: hydrogen bond. Only a subset of binding interactions is shown for clarity. Viewpoint from TM7.

We compared orexin-A C-terminal interactions to suvorexant binding in the OX_2_R crystal structure [13]. Suvorexant binds deep in the cavity with multiple hydrophobic interactions, while the triazole ring is sandwiched within hydrogen-bonding distance between Gln^3.32^ and Asn^6.55^ and the amide carbonyl could hydrogen bond to Asn^6.55^ and His^7.39^ (water-mediated) (Figure 10B). This binding mode does not disturb intramolecular receptor interactions lining the binding pocket, namely Asp^xl2.51^–Arg^6.59^, Glu^xl2.52^–His^5.39^ and Asp^2.65^–His^7.39^.

In our TM7-binding mode, the ligand C-terminus closely follows the hydrogen bonding of suvorexant to Asn^6.55^ and Gln^3.32^ (Figure 10A). The TM7-mode also features His26 close to receptor His344^7.39^ and Asp107^2.65^, which is especially interesting in the light of recent results suggesting that orexin-A binding to OX_1_R is calcium-dependent [50], as histidine/aspartic acid clusters are known to participate in the hexadentate coordination of metal ions. The ligand Leu31 is close enough to break the His216^5.39^–Glu204^xl2.52^ salt bridge. Flexibility and small side chains at the peptide hinge region would permit hydrogen bonds from Asp203^xl2.51^ and Arg322^6.59^ to the peptide backbone. The phenolic oxygen in the Tyr311^6.48^ lies 4.8 Å away from the C-terminal carbonyl, but could reach hydrogen bonding distance with a different rotamer. In other GPCRs, the corresponding Trp^6.48^ is often thought as a key residue for receptor activation.

The TM5-mode, on the other hand, does not replicate the suvorexant hydrogen-bonding pattern (Figure 10C). However, it displays hydrogen bonding to receptor Gln126^3.32^ (ligand T32 carbonyl or C-terminus) and the peptide C-terminus often comes close enough to hydrogen bond to Tyr311^6.48^ (roughly half of the top-ranking poses that adopt the TM5-mode show Tyr311^6.48^ within 4 Å, but the representative pose in Figure 9 does not). The ligand His26 is close to His216^5.39^ and Glu204^xl2.52^, which could serve as a metal binding site as well, and again the hinge region offers hydrogen-bonding sites for Asp203^xl2.51^ and Arg322^6.59^.

These interactions are reminiscent of the activation determinants of other GPCRs. Adrenoceptors, for example, show an active state where the binding site contraction is stabilized by ligand binding between the transmembrane helices, namely hydrogen bonding to Asp^3.32^, Ser^5.42^, Ser^5.46^ and Asn^7.39^ [51-53]. However, even though our binding modes show the orexin peptide deeper than neurotensin in NTSR1, the peptide does not fully reach the depth of the adrenoceptor agonists. Contacts to Phe219^5.42^ are formed by Leu31 in the TM7-mode, and Leu33 in the TM5-mode, but only in few poses within the more open binding cavity of the NTSR1_TM6-based model, the orexin peptide penetrates deep enough to bind to Thr223^5.46^.

Closer to the extracellular surface, the orexin peptide forms interactions which are more like those seen between neurotensin_8–13_ and NTSR1 [35]. Polar ligand residues in the TM7-mode interact with the receptor N-terminus and extracellular loops 1 and 3 (Glu18 with Lys43^1.29^ and Arg333^7.28^, His21 with Glu110^2.68^, Asn25 with Cys202^xl2.50^, and His26 with Asp107^2.65^), whereas in the TM5-mode interaction to the ECL2 and ECL3 take place (His26 to Glu204^xl2.52^, Asn25 to Lys321^6.58^ and Tyr337^7.32^, and His21 to Lys321^6.58^ and to Arg333^7.28^ or Arg328^6.65^). These interactions put together could change the binding site conformation and result in the activation of the receptor.

### Comparison to the receptor point mutations and neurotensin binding

The point-mutation studies on orexin receptors have indicated residues Gln126^3.32^, Val130^3.36^, Asp203^xl2.51^, Trp206^xl2.54^, Tyr215^5.38^, Phe219^5.42^, Thr223^5.46^, Tyr224^5.47^, Tyr311^6.48^, Asn318^6.55^, His344^7.39^ and Tyr348^7.43^ to be relevant for the orexin-peptide-triggered receptor activation (see Background). Of these residues, we already discussed Gln126^3.32^, Asp203^xl2.51^, Phe219^5.42^, Thr223^5.46^, Tyr311^6.48^, Asn318^6.55^ and His344^7.39^ above. Concerning the remaining amino acids, the TM7 binding mode was found to interact also with Tyr215^5.38^ and Tyr348^7.43^ (Figure 8, Table 1). The side chain of Trp206^xl2.54^ lies between TMs 4 and 5, lining the binding cavity, but a different rotamer could bring the side chain closer to the ligand. The side chain of Tyr224^5.47^ lies between TMs 5 and 6 in all models, where it is not exposed to the ligand. Val130^3.36^ at the bottom of the binding cavity is often within 4 Å of the peptide ligand C-terminus. In total, the TM7-mode shows interactions to eight of these residues, whereas the TM5-mode interacts with five.

Residues at the bottom of the binding cavity (Val130^3.36^, Thr223^5.46^, Tyr311^6.48^ and Tyr348^7.43^) are difficult for the ligand to reach in our models. Water molecules are often seen to take part in ligand–receptor interactions, but the applied docking protocol handles water implicitly, so water molecule mediated interactions cannot be addressed. It is also noteworthy that site-directed mutagenesis is an indirect method, and that the indicated residues might not take part directly in the ligand binding, but are part of the receptor activation cascade, or otherwise crucial for the receptor function.

The data from mutation studies was used to direct our docking efforts towards the binding site formed both by the cavity between TM helices and by the loops. This approach has proven effective in the case of neurotensin receptor 1, where extensive mutation and modeling studies had predicted that neurotensin would interact mainly with the ECL3 and upper parts of TMs 6 and 7 [54,55]. The crystal structure was found to be well in line with this prediction [35]. It shows the neurotensin_8–13_ binding in a way consistent with the mutation experiments, fairly high in the cavity (neurotensin_8–13_ Leu13 Cα is ~12 Å above Tyr359^7.43^ Cα). The orexin peptides, however, are considerably larger than neurotensin, and mutational data suggests deeper binding and different interacting residues on the receptor (Figure 3). It is noteworthy that homologous binding-site-facing residues are often smaller in orexin receptors than in NTSR1, permitting the entry of a larger ligand. Changes such as Tyr^3.29^ to proline, Arg^3.32^ to glutamine, Tyr^6.51^ to isoleucine, and Arg^6.55^ to asparagine create a more spacious binding cavity in OX_1_R than observed in NTSR1.

Unfortunately, the mutation studies on the orexin receptors are not as extensive as on the neurotensin receptor 1. Point mutations on orexin receptors have been focused on the residues deep in the cavity, with the exception of few ECL2 residues, whereas the ECL3 has so far been neglected. In addition, in the mutation studies only few residues are reported not to be important, making mutation based comparison and validation of binding modes more difficult.

## Conclusions

In this work, we present two alternative binding modes for orexin-A to OX_1_R, each with their own merits. The receptor models, based on the framework of the neurotensin receptor 1 and the orexin 2 receptor, which was published while this work was under consideration, provide accurate representations of the transmembrane bundle, and the conformation of the extracellular domain. Our docking protocol allows for side chain movements, which should smooth out small-scale inaccuracies in the conformation. The binding modes are consistent with what is known of GPCR activation in general, and fit well to the mutational data. The available mutation data only partially covers the predicted binding site, but we hope our work will direct further mutation studies, especially towards the ECL3. Due to the high sequence identity between the orexin receptor subtypes and similarity of the peptide C-termini, these results should also be transferable to OX_2_R and orexin-B. These alternative binding modes for the orexin-A into OX_1_R, produced by computational modeling and docking, should benefit further characterization of orexin receptor interactions and therapeutic small molecule discovery.

## Endnote

^a^Residues in the transmembrane helices are numbered according to Ballesteros and Weinstein [33]. The most conserved residue of each transmembrane helix is defined as N.50 where N is the ordinal number of the helix counting from the N-terminus. Residues in the ECL2 are numbered similarly so that the bridge-forming cysteine is designated as xl2.50 [56]. In addition, the structure-based residue numbering proposed by the GPCRDB is used when there are differences in the bulges or constrictions within the helices [48].

## Additional files

Additional file 1:
**The conformations of orexin peptides in aqueous solution.** Orexin-A has been reported with multiple conformations that fall into two categories; a bent conformation and a straight conformation. For orexin-B, one conformation has been reported. Additional file 2:
**Structural alignment of crystallized class A GPCRs, and the sequence alignment of human OX**_**1**_ **R.**
Additional file 3:
**The structural alignment from Additional file** **2** **, in FASTA format.** Chain breaks have not been annotated. Additional file 4:
**PDB-file of the OX**_**1**_ **R homology models together with the representative docking poses for the models.** 1: OX_2_R-based model, 2: NTSR1-based model, 3: CXCR4-based model, 4: NTSR1_TM6-based model. Additional file 5:
**Comparison of the secondary models to the OX**_**2**_ **R crystal structure.** Corresponds to Figure 2, but shows the CXCR4- and NTSR1-based models. Additional file 6:
**Additional figures on the docking pose clusters.** Additional figure 6.1: 3D-representations for all docking pose clusters modelwise. AF6.2, AF6.3: Docking pose clusters in the CXCR4- and NTSR1_TM6-based secondary models, corresponding to Figures 4 and 5. AF6.4: Superclustering across models. Additional file 7:
**Statistics for the docking poses, modelwise.** Five plots are shown for each model. 1) A box plot of RDOCK score vs. clusters; 2) Scatter plot of RDOCK score vs. ligand depth; 3) Scatter plot of RDOCK score vs. ligand solvent accessible surface area; 4) Scatter plot of ligand solvent accessible surface area vs. ligand depth; and 5) Distribution of high-scoring poses into clusters. Additional file 8:
**Representative docking poses and interaction heatmaps, modelwise.** Additional figures corresponding to Figures 8 and 9, but for all top-scoring docking poses for each model. Addition figure 8.1 shows the representative high-scoring pose for the OX_2_R-based model, AF8.2 for the NTSR1-based model, AF8.3 for the CXCR4-based model, and AF8.4 for the NTSR1_TM6-based model.

## Abbreviations

TM: Transmembrane segment
GPCR: G protein-coupled receptor
OX_1_R: Orexin receptor 1
OX_2_R: Orexin receptor 2
NTSR1: Neurotensin receptor 1
CXCR4: Chemokine receptor CXCR4
Cα: α carbon
ICL: Intracellular loop
ECL: Extracellular loop
PDB: (RCSB) Protein Data Bank
RMSD: Root-mean-square deviation

## References

[1] Sakurai T, Amemiya A, Ishii M, Matsuzaki I, Chemelli RM, Tanaka H (1998). Orexins and orexin receptors: a family of hypothalamic neuropeptides and G protein-coupled receptors that regulate feeding behavior. Cell.

[2] Sakurai T, Mieda M, Tsujino N (2010). The orexin system: roles in sleep/wake regulation. Ann N Y Acad Sci.

[3] Laburthe M, Voisin T (2012). The orexin receptor OX1R in colon cancer: a promising therapeutic target and a new paradigm in G protein-coupled receptor signalling through ITIMs. Br J Pharmacol.

[4] Scammell TE, Winrow CJ (2011). Orexin receptors: pharmacology and therapeutic opportunities. Annu Rev Pharmacol Toxicol.

[5] Haynes AC, Jackson B, Chapman H, Tadayyon M, Johns A, Porter RA (2000). A selective orexin-1 receptor antagonist reduces food consumption in male and female rats. Regul Pept.

[6] De Lecea L, Kilduff TS, Peyron C, Gao X-B, Foye PE, Danielson PE (1998). The hypocretins: hypothalamus-specific peptides with neuroexcitatory activity. Proc Natl Acad Sci U S A.

[7] Lee J-H, Bang E, Chae K-J, Kim J-Y, Lee DW, Lee W (1999). Solution structure of a new hypothalamic neuropeptide, human hypocretin-2/orexin-B. Eur J Biochem.

[8] Takai T, Takaya T, Nakano M, Akutsu H, Nakagawa A, Aimoto S (2006). Orexin-A is composed of a highly conserved C-terminal and a specific, hydrophilic N-terminal region, revealing the structural basis of specific recognition by the orexin-1 receptor. J Pept Sci.

[9] Darker JG, Porter RA, Eggleston DS, Smart D, Brough SJ, Sabido-David C (2001). Structure-activity analysis of truncated orexin-A analogues at the orexin-1 receptor. Bioorg Med Chem Lett.

[10] Ammoun S, Holmqvist T, Shariatmadari R, Oonk HB, Detheux M, Parmentier M (2003). Distinct recognition of OX1 and OX2 receptors by orexin peptides. J Pharmacol Exp Ther.

[11] Lang M, Söll RM, Dürrenberger F, Dautzenberg FM, Beck-Sickinger AG (2004). Structure-activity studies of orexin A and orexin B at the human orexin 1 and orexin 2 receptors led to orexin 2 receptor selective and orexin 1 receptor preferring. J Med Chem.

[12] Asahi S, Egashira S-I, Matsuda M, Iwaasa H, Kanatani A, Ohkubo M (2003). Development of an orexin-2 receptor selective agonist, [Ala (11), D-Leu (15)] orexin-B. Bioorg Med Chem Lett.

[13] Yin J, Mobarec JC, Kolb P, Rosenbaum DM (2015). Crystal structure of the human OX2 orexin receptor bound to the insomnia drug suvorexant. Nature.

[14] Malherbe P, Roche O, Marcuz A, Kratzeisen C, Wettstein JG, Bissantz C (2010). Mapping the binding pocket of dual antagonist almorexant to human orexin 1 and Orexin 2 receptors: comparison with the selective OX1 antagonist SB-674042 and the selective OX2 antagonist N-Ethyl-2-[(6-methoxy-pyridin-3-yl)-(toluene-2-sulfonyl)-amino]-N-py. Mol Pharmacol.

[15] Tran D-T, Bonaventure P, Hack M, Mirzadegan T, Dvorak C, Letavic M (2011). Chimeric, mutant orexin receptors show key interactions between orexin receptors, peptides and antagonists. Eur J Pharmacol.

[16] Putula J, Kukkonen JP (2012). Mapping of the binding sites for the OX1 orexin receptor antagonist, SB-334867, using orexin/hypocretin receptor chimaeras. Neurosci Lett.

[17] Michino M, Abola E, Brooks CL, Dixon JS, Moult J, GPCR Dock 2008 participants (2009). Community-wide assessment of GPCR structure modelling and ligand docking: GPCR Dock 2008. Nat Rev Drug Discov.

[18] Kufareva I, Rueda M, Katritch V, Stevens RC, Abagyan R, GPCR Dock 2010 participants (2011). Status of GPCR modeling and docking as reflected by community-wide GPCR Dock 2010 assessment. Structure.

[19] Kufareva I, Katritch V, Stevens RC, Abagyan R, Participants of GPCR Dock 2013 (2014). Advances in GPCR modeling evaluated by the GPCR dock 2013 assessment: meeting new challenges. Structure.

[20] Trellet M, Melquiond ASJ, Bonvin AMJJ (2013). A unified conformational selection and induced fit approach to protein-peptide docking. PLoS One.

[21] Raveh B, London N, Schueler-Furman O (2010). Sub-angstrom modeling of complexes between flexible peptides and globular proteins. Proteins.

[22] Antes I (2010). DynaDock: a new molecular dynamics-based algorithm for protein-peptide docking including receptor flexibility. Proteins.

[23] Prusis P, Schiöth HB, Muceniece R, Herzyk P, Afshar M, Hubbard RE (1997). Modeling of the three-dimensional structure of the human melanocortin 1 receptor, using an automated method and docking of a rigid cyclic melanocyte-stimulating hormone core peptide. J Mol Graph Model.

[24] De Wachter R, De Graaf C, Keresztes A, Vandormael B, Ballet S, Rognan D (2011). Synthesis, biological evaluation, and automated docking of constrained analogues of the opioid peptide H-Dmt- D -Ala-Phe-Gly- 5-tetrahydro-2-benzazepin-3-one Scaffold. J Med Chem.

[25] Chandrashekaran IR, Rao GS, Cowsik SM (2009). Molecular modeling of the peptide agonist-binding site in a neurokinin-2 receptor. J Chem Inf Model.

[26] Ganjiwale AD, Rao GS, Cowsik SM (2011). Molecular modeling of neurokinin B and tachykinin NK_3_ receptor complex. J Chem Inf Model.

[27] Matsoukas M-T, Potamitis C, Plotas P, Androutsou M, Agelis G, Matsoukas J (2013). Insights into AT1 receptor activation through AngII binding studies. J Chem Inf Model.

[28] Heifetz A, Barker O, Morris GB, Law RJ, Slack M, Biggin PC (2013). Toward an understanding of agonist binding to human Orexin-1 and Orexin-2 receptors with G-protein-coupled receptor modeling and site-directed mutagenesis. Biochem.

[29] Rodrigo J, Pena A, Murat B, Trueba M, Durroux T, Guillon G (2007). Mapping the binding site of Arginine Vasopressin to V 1a and V 1b Vasopressin receptors. Mol Endocrinol.

[30] Chen R, Li L, Weng Z (2003). ZDOCK: an initial-stage protein-docking algorithm. Proteins.

[31] Li L, Chen R, Weng Z (2003). RDOCK: refinement of rigid-body protein docking predictions. Proteins.

[32] Discovery Studio (2012). Version 3.5.

[33] Ballesteros JA, Weinstein H (1995). Integrated methods for the construction of three-dimensional models and computational probing of structure-function relations in G protein-coupled receptors. Methods Neurosci.

[34] Wu B, Chien EYT, Mol CD, Fenalti G, Liu W, Katritch V (2010). Structures of the CXCR4 chemokine GPCR with small-molecule and cyclic peptide antagonists. Science.

[35] White JF, Noinaj N, Shibata Y, Love J, Kloss B, Xu F (2012). Structure of the agonist-bound neurotensin receptor. Nature.

[36] Manglik A, Kruse AC, Kobilka TS, Thian FS, Mathiesen JM, Sunahara RK (2012). Crystal structure of the μ-opioid receptor bound to a morphinan antagonist. Nature.

[37] Wu H, Wacker D, Mileni M, Katritch V, Han GW, Vardy E (2012). Structure of the human κ-opioid receptor in complex with JDTic. Nature.

[38] Granier S, Manglik A, Kruse AC, Kobilka TS, Thian FS, Weis WI (2012). Structure of the δ-opioid receptor bound to naltrindole. Nature.

[39] Thompson AA, Liu W, Chun E, Katritch V, Wu H, Vardy E (2012). Structure of the nociceptin/orphanin FQ receptor in complex with a peptide mimetic. Nature.

[40] Fredriksson R, Lagerström MC, Lundin L-G, Schiöth HB (2003). The G-protein-coupled receptors in the human genome form five main families: phylogenetic analysis, paralogon groups, and fingerprints. Mol Pharmacol.

[41] Maestro (2013). Version 9.4.

[42] Chien EYT, Liu W, Zhao Q, Katritch V, Han GW, Hanson MA (2010). Structure of the human dopamine D3 receptor in complex with a D2/D3 selective antagonist. Science.

[43] Egloff P, Hillenbrand M, Klenk C, Batyuk A, Heine P, Balada S (2014). Structure of signaling-competent neurotensin receptor 1 obtained by directed evolution in Escherichia coli. Proc Natl Acad Sci U S A.

[44] Săli A, Blundell TL (1993). Comparative protein modelling by satisfaction of spatial restraints. J Mol Biol.

[45] Daura X, Gademann K, Jaun B, Seebach D, Van Gunsteren WF, Mark AE (1999). Peptide folding: when simulation meets experiment. Angew Chemie Int Ed.

[46] Matlab (2013). Version R2013a.

[47] Hubbard SJ, Thornton JM (1993). Naccess.

[48] Isberg V, de Graaf C, Bortolato A, Cherezov V, Katritch V, Marshall FH (2015). Generic GPCR residue numbers – aligning topology maps while minding the gaps. Trends Pharmacol Sci.

[49] Voss NR, Gerstein M (2010). 3V: cavity, channel and cleft volume calculator and extractor. Nucleic Acids Res.

[50] Putula J, Pihlajamaa T, Kukkonen JP (2014). Calcium affects OX1 orexin (hypocretin) receptor responses by modifying both orexin binding and the signal transduction machinery. Br J Pharmacol.

[51] Warne T, Moukhametzianov R, Baker JG, Nehme R, Edwards PC, Leslie AGW (2011). The structural basis for agonist and partial agonist action on a β1-adrenergic receptor. Nature.

[52] Rasmussen SGF, Choi H-J, Fung JJ, Pardon E, Casarosa P, Chae PS (2011). Structure of a nanobody-stabilized active state of the β(2) adrenoceptor. Nature.

[53] Xhaard H, Rantanen V-V, Nyronen T, Johnson MS (2006). Molecular evolution of adrenoceptors and dopamine receptors: implications for the binding of catecholamines. J Med Chem.

[54] Kitabgi P (2006). Functional domains of the subtype 1 neurotensin receptor (NTS1). Peptides.

[55] Härterich S, Koschatzky S, Einsiedel J, Gmeiner P (2008). Novel insights into GPCR-peptide interactions: mutations in extracellular loop 1, ligand backbone methylations and molecular modeling of neurotensin receptor 1. Bioorg Med Chem.

[56] Xhaard H, Nyrönen T, Rantanen V-V, Ruuskanen JO, Laurila J, Salminen T (2005). Model structures of α2-adrenoceptors in complex with automatically docked antagonist ligands raise the possibility of interactions dissimilar from agonist ligands. J Struct Biol.

[57] Barton GJ (1993). ALSCRIPT: a tool to format multiple sequence alignments. Protein Eng.

