# Supplementary material for: Modeling of the OX1R–orexin-A complex suggests two alternative binding modes
Source: BMC Struct Biol. 2015 May 9;15:9. doi: 10.1186/s12900-015-0036-2 (PMC4469407; doi:10.1186/s12900-015-0036-2)
Supplement: Additional file 1: — The conformations of orexin peptides in aqueous solution. Orexin-A has been reported with multiple conformations that fall into two categories; a bent conformation and a straight conformation. For orexin-B, one conformation has been reported. [file 12900_2015_36_MOESM1_ESM.pdf]

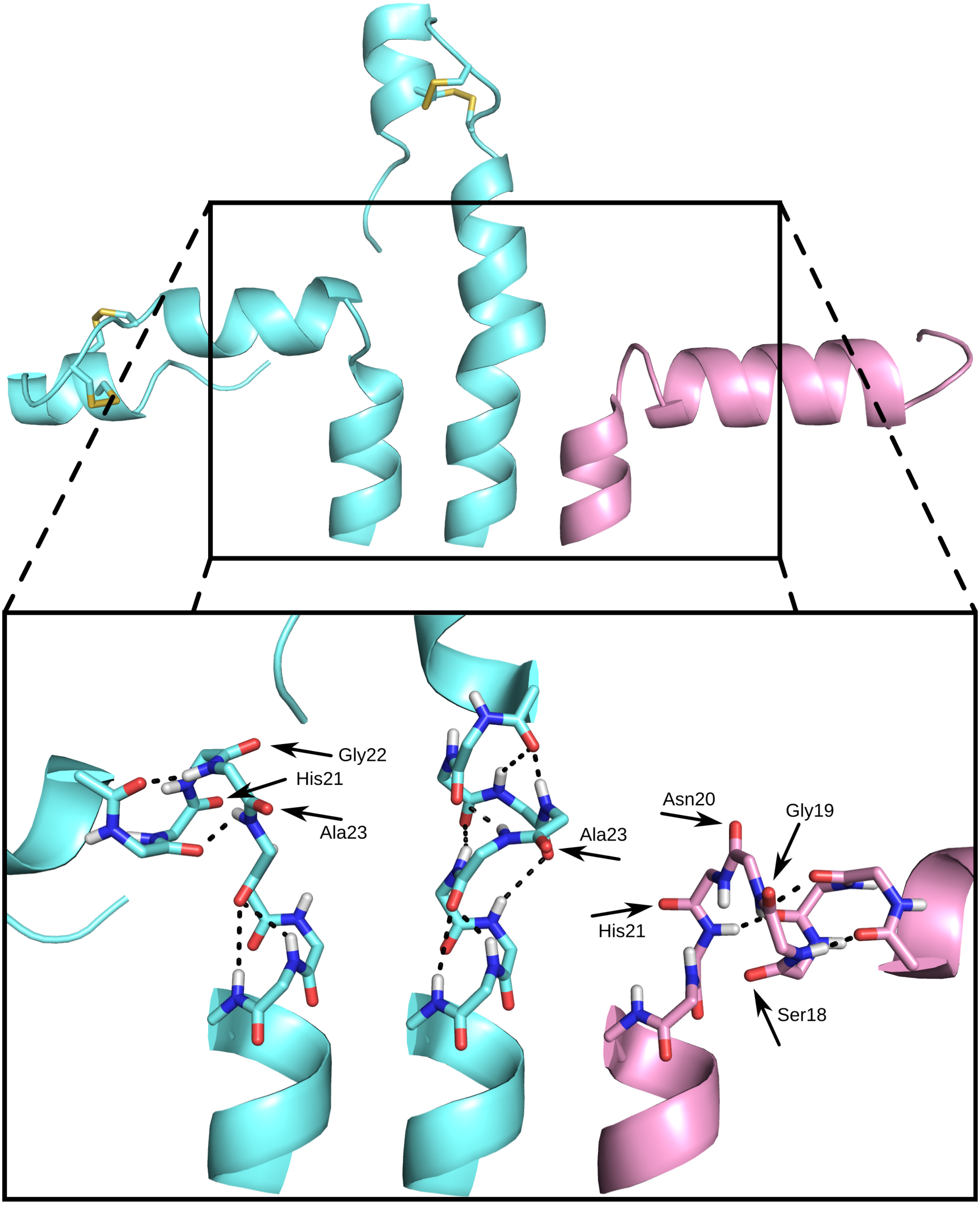


**Additional figure 1:** The conformations of orexin-A (cyan) and orexin-B (pink) in aqueous solution. Takai and co-workers reported 30 models of the orexin-A structure [1], which can be divided into two distinct groups; those in bent conformation (25/30, left in the figure) and those in straight conformation (5/30, middle). Orexin-B adopts a bent conformation different from the conformation seen in orexin-A [2]. These conformations differ in the hinge region, leading to different number of hydrogen bond acceptor exposed to solvent (arrows).

References:

1. Takai T, Takaya T, Nakano M, Akutsu H, Nakagawa A, Aimoto S, Nagai K, Ikegami T: **Orexin-A is composed of a highly conserved C-terminal and a specific, hydrophilic N-terminal region, revealing the structural basis of specific recognition by the orexin-1 receptor.** *J Pept Sci* 2006, **12**:443–454.

2. Lee J-H, Bang E, Chae K-J, Kim J-Y, Lee DW, Lee W: **Solution structure of a new hypothalamic neuropeptide, human hypocretin-2/orexin-B.** *Eur J Biochem* 1999, **266**:831–839.
