# Supplementary material for: Modeling of the OX1R–orexin-A complex suggests two alternative binding modes
Source: BMC Struct Biol. 2015 May 9;15:9. doi: 10.1186/s12900-015-0036-2 (PMC4469407; doi:10.1186/s12900-015-0036-2)
Supplement: Additional file 2: — Structural alignment of crystallized class A GPCRs, and the sequence alignment of human OX 1 R. [file 12900_2015_36_MOESM2_ESM.pdf]

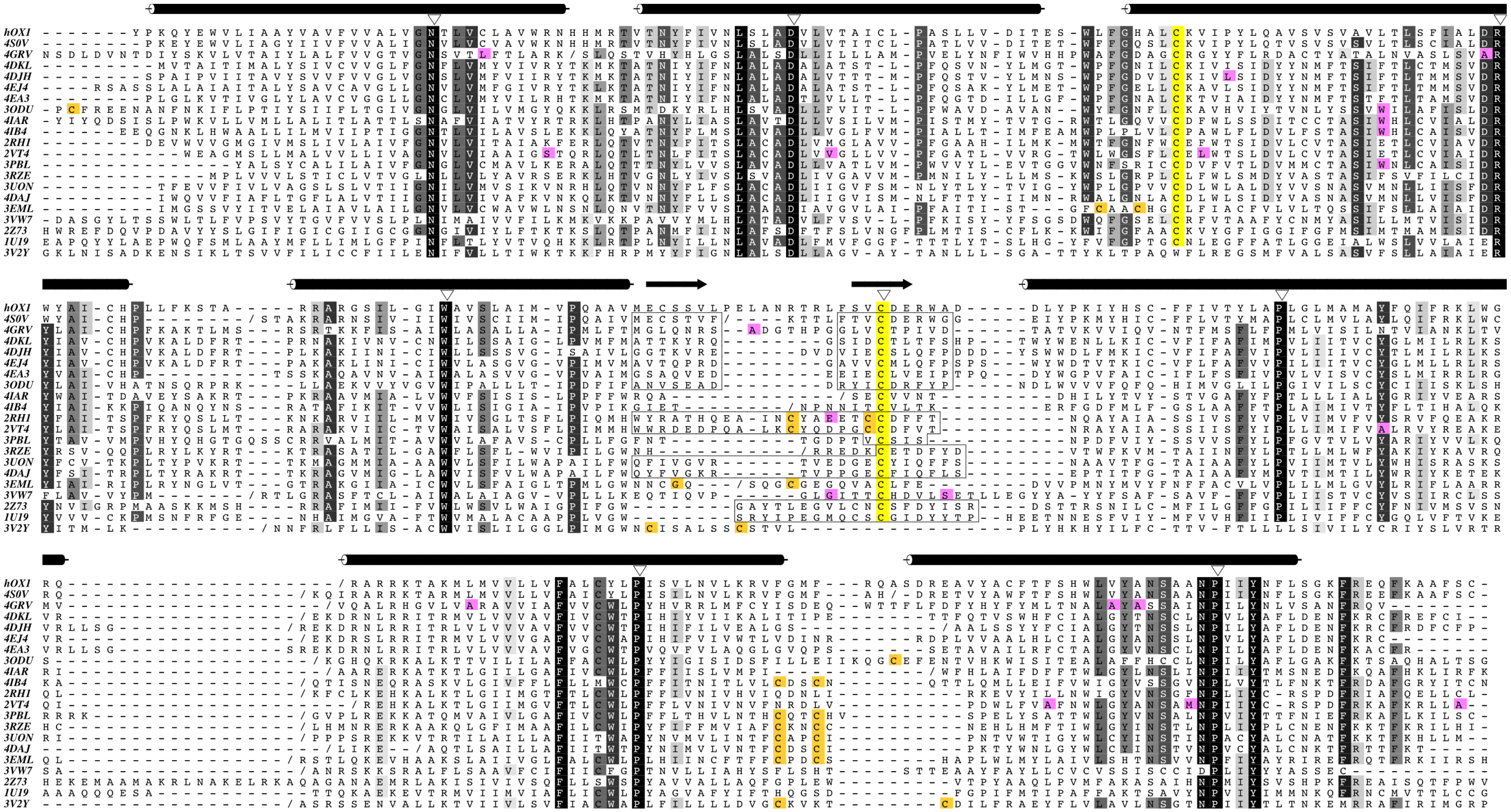
hOX1
4S0V
4GRV
4DKL
4DJH
4EJ4
4EA3
3ODU
4IAR
4IB4
2RH1
2VT4
3PBL
3RZE
3UON
4DAJ
3EML
3VW7
2Z73
1U19
3V2Y

Additional figure 2: Structural alignment of crystallized GPCRs from class A, and the sequence alignment of human OX_1R. Yellow/orange: disulfide bridge. Magenta: mutated residue (for increased stability or expression). Cylinder: approximate location of transmembrane helices. Arrow: β strands in the ECL2 of peptide binding receptors. Boxed regions: structural alignment of a subset of receptors. Illustrated with Alscript [1]

PDB identifiers:

4S0V, orexin receptor 2 [2]
4GRV, rat neurotensin 1 [3]
4DKL, mouse mu opioid [4]
4DJH, kappa opioid [5]
4EJ4, mouse delta opioid [6]
4EA3, nociceptin [7]
3ODU, chemokine CXCR4 [8]
4IAR, serotonin 5-$HT_{1}B$ [9]
4IB4, serotonin 5-$HT_{2}B$ [10]
2RH1, β_2-adrenoceptor [11]
2VT4, turkey β_1-adrenoceptor [12]
3PBL, dopamine D_3 [13]
3RZE, histamine H1 [14]
3UON, M2 muscarinic [15]
4DAJ, rat M3 muscarinic [16]
3EML, adenosine A_{2A} [17]
3VW7, proteinase-activated receptor-1 (PAR1) [18]
2Z73, squid rhodopsin [19]
1U19, bovine rhodopsin [20]
3V2Y, sphingosine 1-phosphate 1 [21]

References:

1. Barton GJ: **ALSCRIPT: a tool to format multiple sequence alignments**. *Protein Eng* 1993, **6**:37–40.

2. Yin J, Mobarec JC, Kolb P, Rosenbaum DM: **Crystal structure of the human OX2 orexin receptor bound to the insomnia drug suvorexant**. *Nature* 2014.

3. White JF, Noinaj N, Shibata Y, Love J, Kloss B, Xu F, Gvozdenovic-Jeremic J, Shah P, Shiloach J, Tate CG, Grisshammer R: **Structure of the agonist-bound neurotensin receptor.** *Nature* 2012, **490**:508–513.

4. Manglik A, Kruse AC, Kobilka TS, Thian FS, Mathiesen JM, Sunahara RK, Pardo L, Weis WI, Kobilka BK, Granier S: **Crystal structure of the μ-opioid receptor bound to a morphinan antagonist**. *Nature* 2012, **485**:321–326.

5. Wu H, Wacker D, Mileni M, Katritch V, Han GW, Vardy E, Liu W, Thompson A a, Huang X-P, Carroll FI, Mascarella SW, Westkaemper RB, Mosier PD, Roth BL, Cherezov V, Stevens RC: **Structure of the human κ-opioid receptor in complex with JDTic.** *Nature* 2012, **485**:327–332.

6. Granier S, Manglik A, Kruse AC, Kobilka TS, Thian FS, Weis WI, Kobilka BK: **Structure of the δ-opioid receptor bound to naltrindole.** *Nature* 2012, **485**:400–404.

7. Thompson A a, Liu W, Chun E, Katritch V, Wu H, Vardy E, Huang X-P, Trapella C, Guerrini R, Calo G, Roth BL, Cherezov V, Stevens RC: **Structure of the nociceptin/orphanin FQ receptor in complex with a peptide mimetic.** *Nature* 2012, **485**:395–399.

8. Wu B, Chien EYT, Mol CD, Fenalti G, Liu W, Katritch V, Abagyan R, Brooun A, Wells P, Bi FC, Hamel DJ, Kuhn P, Handel TM, Cherezov V, Stevens RC: **Structures of the CXCR4 chemokine GPCR with small-molecule and cyclic peptide antagonists.** *Science* 2010, **330**:1066–1071.

9. Wang C, Jiang Y, Ma J, Wu H, Wacker D, Katritch V, Han GW, Liu W, Huang X-P, Vardy E, McCorvy JD, Gao X, Zhou XE, Melcher K, Zhang C, Bai F, Yang H, Yang L, Jiang H, Roth BL, Cherezov V, Stevens RC, Xu HE: **Structural basis for molecular recognition at serotonin receptors.** *Science* 2013, **340**:610–614.

10. Wacker D, Wang C, Katritch V, Han GW, Huang X-P, Vardy E, McCorvy JD, Jiang Y, Chu M, Siu FY, Liu W, Xu HE, Cherezov V, Roth BL, Stevens RC: **Structural features for functional selectivity at serotonin receptors.** *Science* 2013, **340**:615–619.

11. Cherezov V, Rosenbaum DM, Hanson M a, Rasmussen SGF, Thian FS, Kobilka TS, Choi H-J, Kuhn P, Weis WI, Kobilka BK, Stevens RC: **High-resolution crystal structure of an engineered human beta2-adrenergic G protein-coupled receptor.** *Science* 2007, **318**:1258–1265.

12. Warne T, Serrano-Vega MJ, Baker JG, Moukhametzianov R, Edwards PC, Henderson R, Leslie AGW, Tate CG, Schertler GFX: **Structure of a beta1-adrenergic G-protein-coupled receptor.** *Nature* 2008, **454**:486–491.

13. Chien EYT, Liu W, Zhao Q, Katritch V, Han GW, Hanson MA, Shi L, Newman AH, Javitch JA, Cherezov V, Stevens RC: **Structure of the human dopamine D3 receptor in complex with a D2/D3 selective antagonistStructure of the human dopamine D3 receptor in complex with a D2/D3 selective antagonist**. *Science* 2010, **330**:1091–1905.

14. Shimamura T, Shiroishi M, Weyand S, Tsujimoto H, Winter G, Katritch V, Abagyan R, Cherezov V, Liu W, Han GW, Kobayashi T, Stevens RC, Iwata S: **Structure of the human histamine H1 receptor complex with doxepin**. *Nature* 2011, **475**:65–72.

15. Haga K, Kruse AC, Asada H, Yurugi-Kobayashi T, Shiroishi M, Zhang C, Weis WI, Okada T, Kobilka BK, Haga T, Kobayashi T: **Structure of the human M2 muscarinic acetylcholine receptor bound to an antagonist.** *Nature* 2012, **482**:547–551.

16. Kruse AC, Hu J, Pan AC, Arlow DH, Rosenbaum DM, Rosemond E, Green HF, Liu T, Chae PS, Dror RO, Shaw DE, Weis WI, Wess J, Kobilka BK: **Structure and dynamics of the M3 muscarinic acetylcholine receptor.** *Nature* 2012, **482**:552–556.

17. Jaakola V-P, Griffith MT, Hanson MA, Cherezov V, Chien EYT, Lane JR, IJzerman AP, Stevens RC: **The 2.6 angstrom crystal structure of a human A2A adenosine receptor bound to an antagonist**. *Science* 2008, **322**:1211–1217.

18. Zhang C, Srinivasan Y, Arlow DH, Fung JJ, Palmer D, Zheng Y, Green HF, Pandey A, Dror RO, Shaw DE, Weis WI, Coughlin SR, Kobilka BK: **High-resolution crystal structure of human protease-activated receptor 1.** *Nature* 2012, **492**:387–392.

19. Murakami M, Kouyama T: **Crystal structure of squid rhodopsin.** *Nature* 2008, **453**:363–367.

20. Okada T, Sugihara M, Bondar A-N, Elstner M, Entel P, Buss V: **The retinal conformation and its environment in rhodopsin in light of a new 2.2 A crystal structure.** *J Mol Biol* 2004, **342**:571–583.

21. Hanson MA, Roth CB, Jo E, Griffith MT, Scott FL, Reinhart G, Desale H, Clemons B, Cahalan SM, Schuerer SC, Sanna MG, Han GW, Kuhn P, Rosen H, Stevens RC: **Crystal structure of a lipid G protein-coupled receptor.** *Science* 2012, **335**:851–855.
