# Supplementary material for: Modeling of the OX1R–orexin-A complex suggests two alternative binding modes
Source: BMC Struct Biol. 2015 May 9;15:9. doi: 10.1186/s12900-015-0036-2 (PMC4469407; doi:10.1186/s12900-015-0036-2)
Supplement: Additional file 5: — Comparison of the secondary models to the OX 2 R crystal structure. Corresponds to Figure 2, but shows the CXCR4- and NTSR1-based models. [file 12900_2015_36_MOESM5_ESM.pdf]

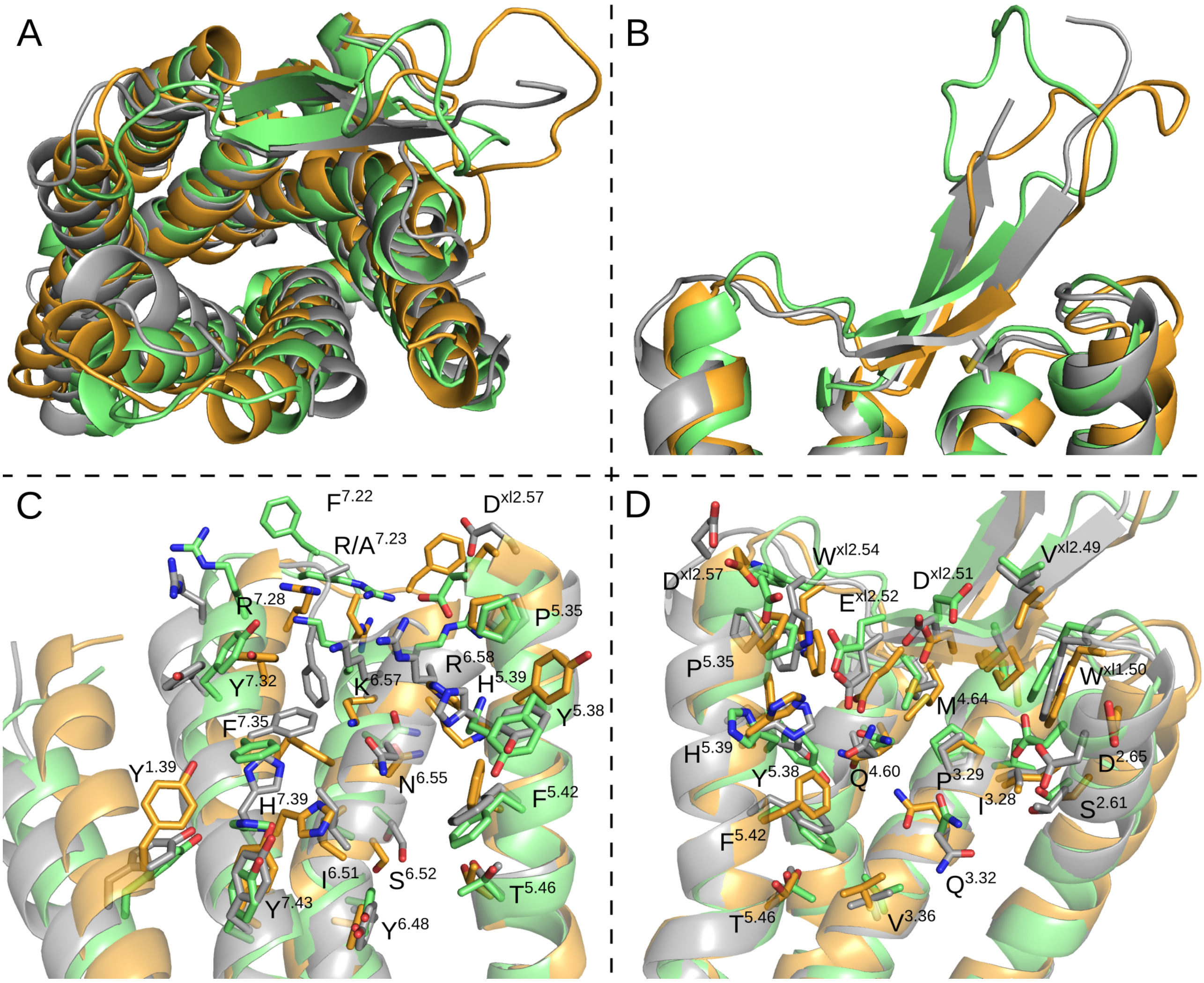

**Additional figure 5:** Comparison of the secondary models to the $OX_2R$ crystal structure, corresponding to Figure 3 in the article. Orange: CXCR4-based model. Green: NTSR1_TM6-based model. Gray: $OX_2R$ crystal structure. (A) Extracellular view shows that the overall structure is fairly similar except for the TM6 and the extracellular end of TM7. (B) The conformation of ECL2. (C and D) The binding-site-facing residues from TMs 1, 5–7, and TMs 2–5 respectively.
