# Supplementary material for: Modeling of the OX1R–orexin-A complex suggests two alternative binding modes
Source: BMC Struct Biol. 2015 May 9;15:9. doi: 10.1186/s12900-015-0036-2 (PMC4469407; doi:10.1186/s12900-015-0036-2)
Supplement: Additional file 6: — Additional figures on the docking pose clusters. Additional figure 6.1: 3D-representations for all docking pose clusters modelwise. AF6.2, AF6.3: Docking pose clusters in the CXCR4- and NTSR1_TM6-based secondary models, corresponding to Figures 4 and 5. AF6.4: Superclustering across models. [file 12900_2015_36_MOESM6_ESM.pdf]

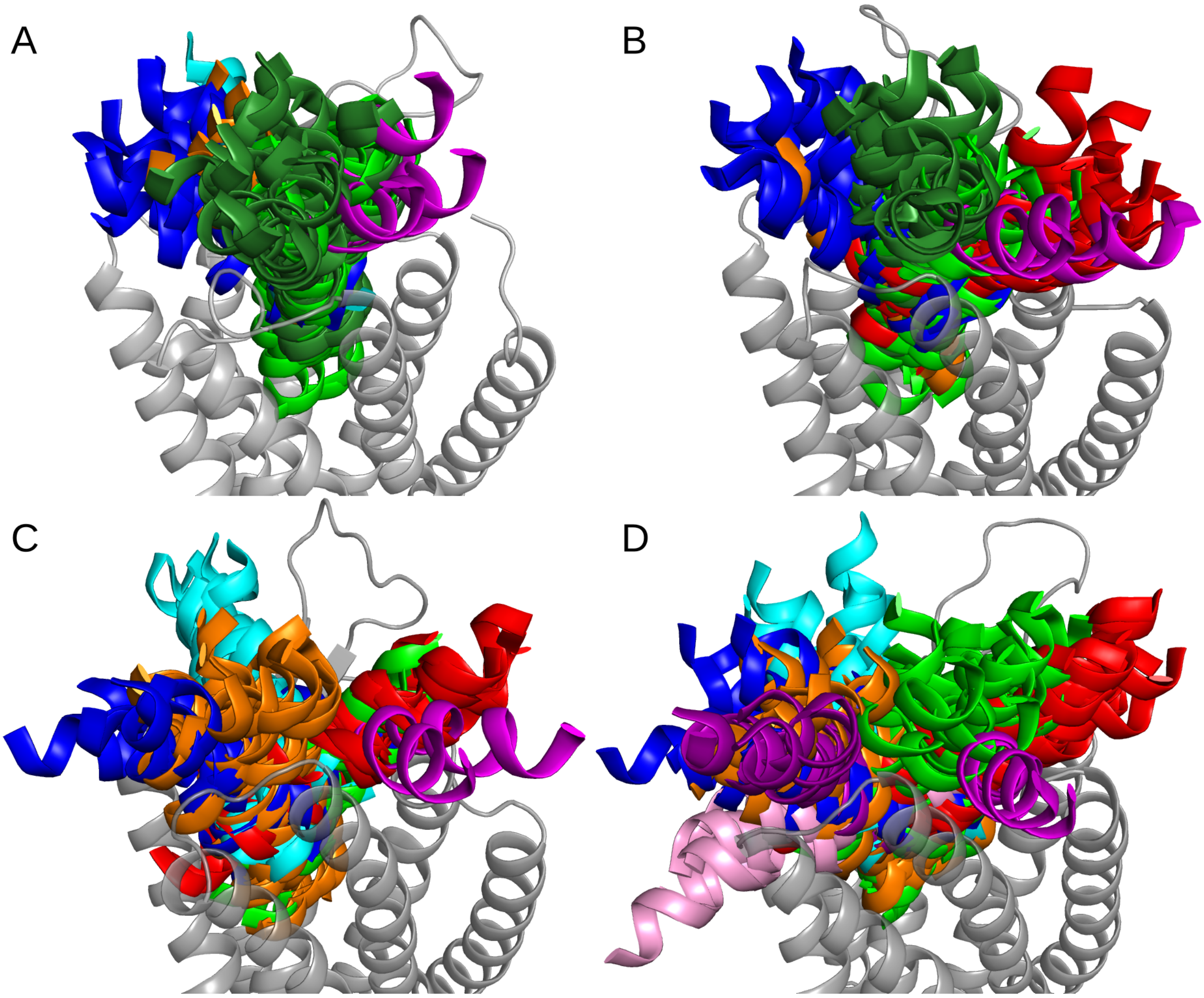

Additional figure 6.1: 3D-representation for all docking pose clusters, modelwise. (A) OX_2R-based model, (B) NTSR1-based model, (C) NTSR1_TM6-based model, (D) CXCR4-based model.

Poses leaning towards the TMs 1–2 are shown in shades of red/magenta, poses leaning to the TMs 5–6 are cyan, blue or purple, and poses vertically in the cavity are orange, green or dark green.

See Additional figure 6.4 for more information on coloring.

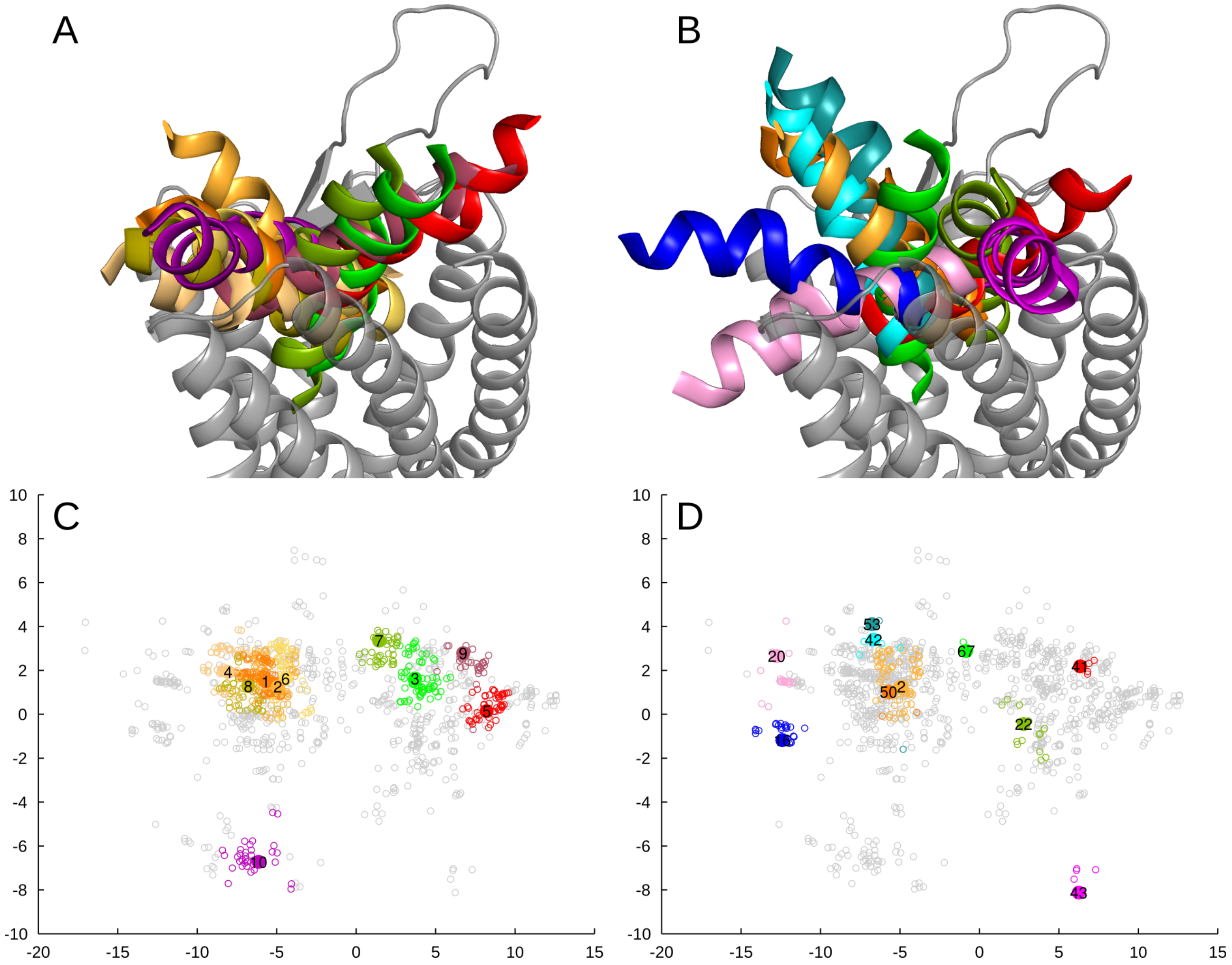

Additional figure 6.2: Corresponding to Figures 4–5 in the article, 3D-representations for the docking pose clusters and scatter plots from multidimensional scaling, CXCR4-based model. (A, C) Ten largest clusters; (B, D) Ten top-scoring clusters. In panels A–B, the TM1 is on the right. Multidimensional scaling shows the clusters (colored; numbers refer to size ranking) in respect to the pool of docking poses (gray).

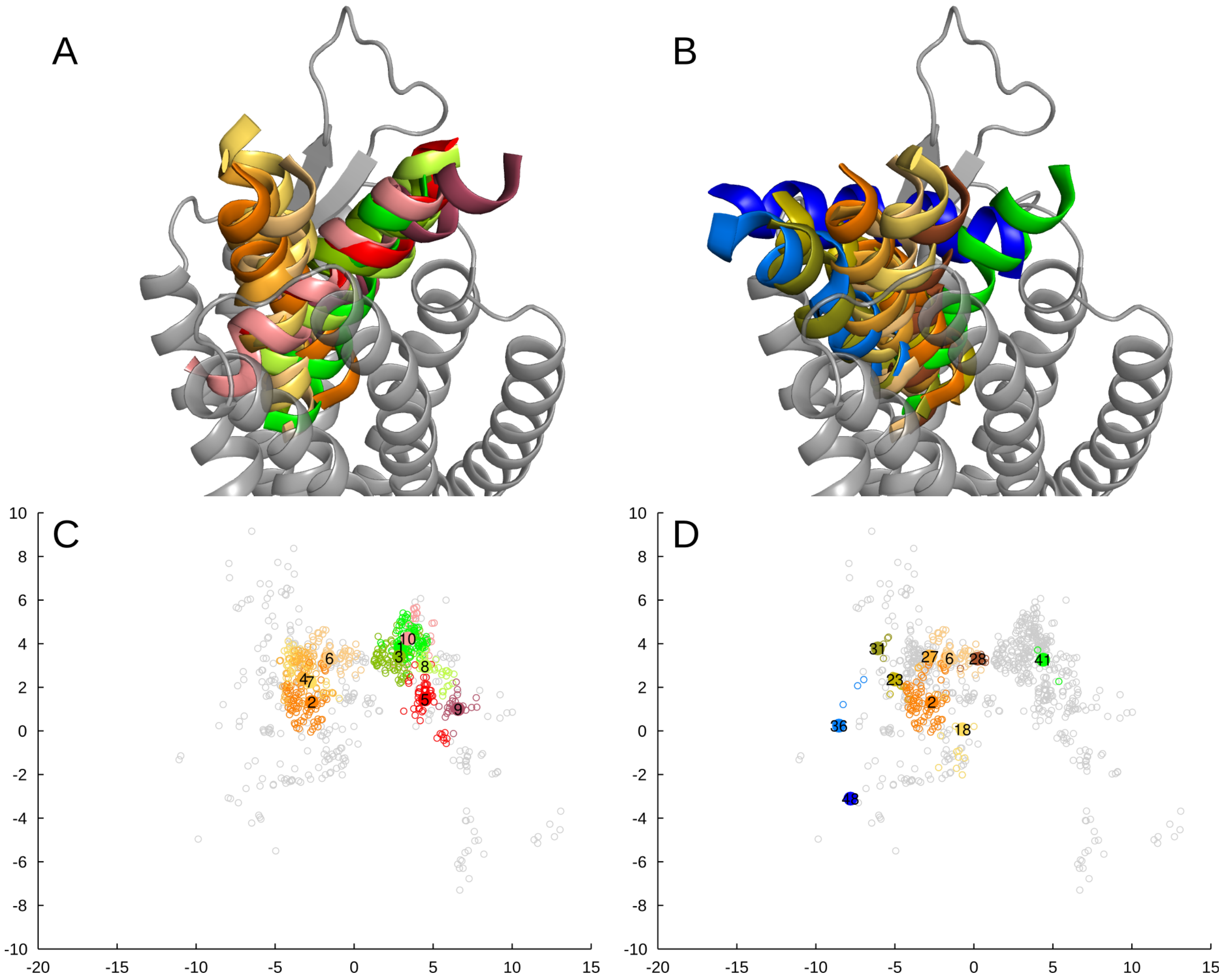

Additional figure 6.3: 3D-representations for the docking pose clusters and scatter plots from multidimensional scaling, NTSR1_TM6-based model. See AF6.2 for full legend.

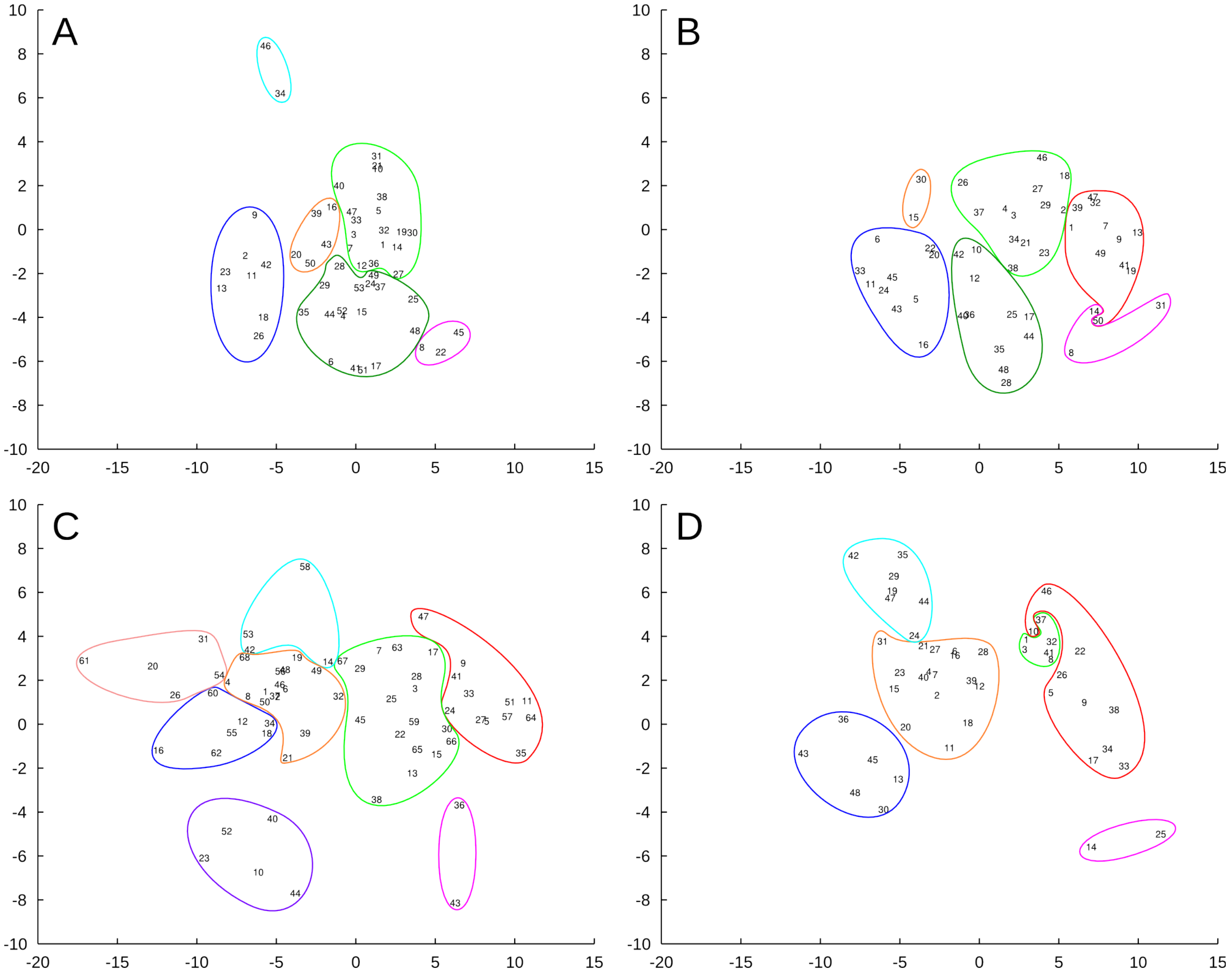

Additional figure 6.4: Multidimensional scaling and superclustering modelwise.
(A) OX$_2$R-based model, (B) NTSR1-based model, (C) CXCR4-based model,
(D) NTSR1_TM6-based model.

Clusters occupying the same area of the binding cavity were grouped; illustrated here with perimeters of different colors. The coloring is consistent with 3D-representations of cluster seeds (Figures 4–5 and all figures within this file). Red: Slanted to TM1. Magenta: Slanted to TM2. Purple: Vertical, high, slanted to ECL3. Blue: Slanted to TM5, medium high. Pink: Horizontal, between TMs 5 and 6. Cyan: Slightly slanted to ECL2, medium deep. Dark green: Vertical, center of the cavity, medium deep. Green: Vertical, center of the cavity, deep. Orange: Vertical, deep, TM5-side of cavity.
