# Supplementary material for: Modeling of the OX1R–orexin-A complex suggests two alternative binding modes
Source: BMC Struct Biol. 2015 May 9;15:9. doi: 10.1186/s12900-015-0036-2 (PMC4469407; doi:10.1186/s12900-015-0036-2)
Supplement: Additional file 7: — Statistics for the docking poses, modelwise. Five plots are shown for each model. 1) A box plot of RDOCK score vs. clusters; 2) Scatter plot of RDOCK score vs. ligand depth; 3) Scatter plot of RDOCK score vs. ligand solvent accessible surface area; 4) Scatter plot of ligand solvent accessible surface area vs. ligand depth; and 5) Distribution of high-scoring poses into clusters. [file 12900_2015_36_MOESM7_ESM.pdf]

Additional file 7: Modelwise statistics on docking results.

Figure 7.1: OX_2R-based model, Figure 7.2: NTSR1-based model
Figure 7.3: CXCR4-based model, Figure 7.4: NTSR1_TM6-based model

For each model, there are the following plots:

1) Box plot of RDOCK scores as a function of cluster. Box shows 25-75%, whiskers extend to non-outliers. Outliers are shown as red '+', median is shown as a red line.

2) Scatter plot of the RDOCK score as a function of ligand binding depth (measured as the Z-coordinate of the ligand N-terminal Leu33 α carbon). Zero-depth is set to the plane of α carbons from $Thr223^{5.46}$, $Tyr311^{6.48}$ and $Tyr348^{7.43}$.

3) Scatter plot of the RDOCK score as a function of ligand solvent accessible surface area.

4) Scatter plot of the ligand solvent accessible surface area as a function of binding depth.

5) Clusterwise histogram of the distribution of high scoring poses. Red: Top 5% of the poses, Blue: Top 10%. Above each bar, the fraction of top 10 % ranking poses of the total cluster size is shown.

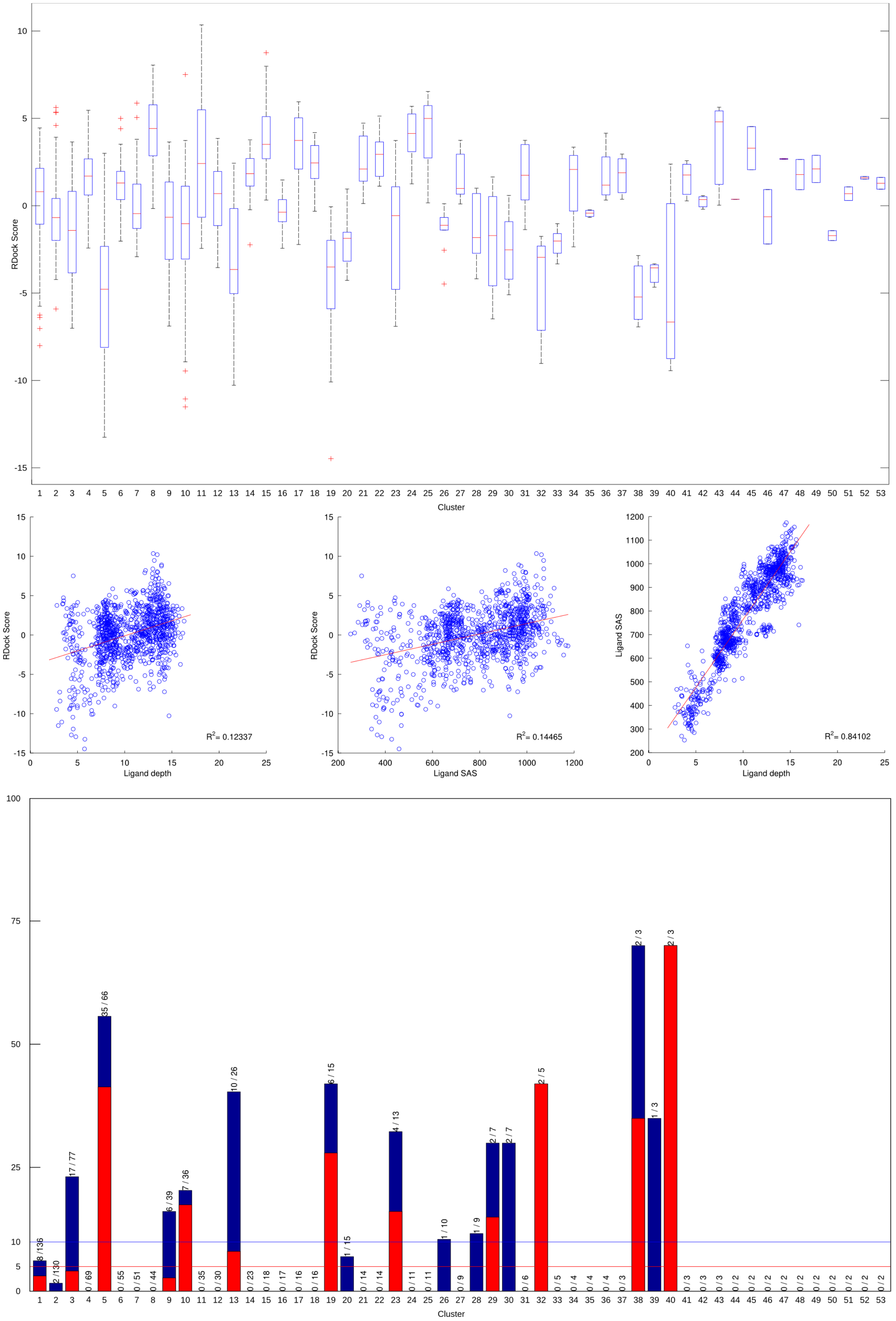

Additional figure 7.1: Docking result statistics for the OX_2R-based model.

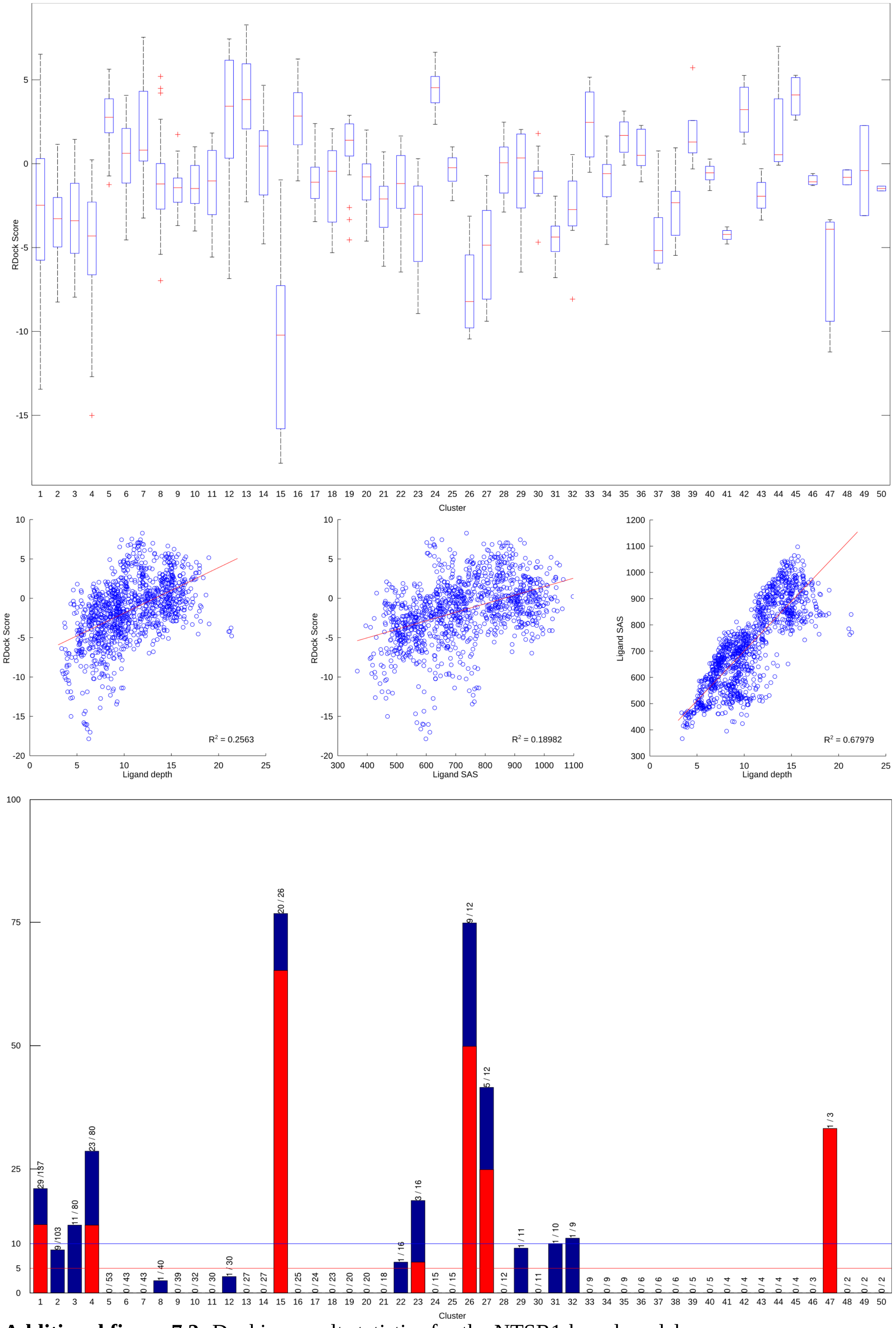

Additional figure 7.2: Docking result statistics for the NTSR1-based model.

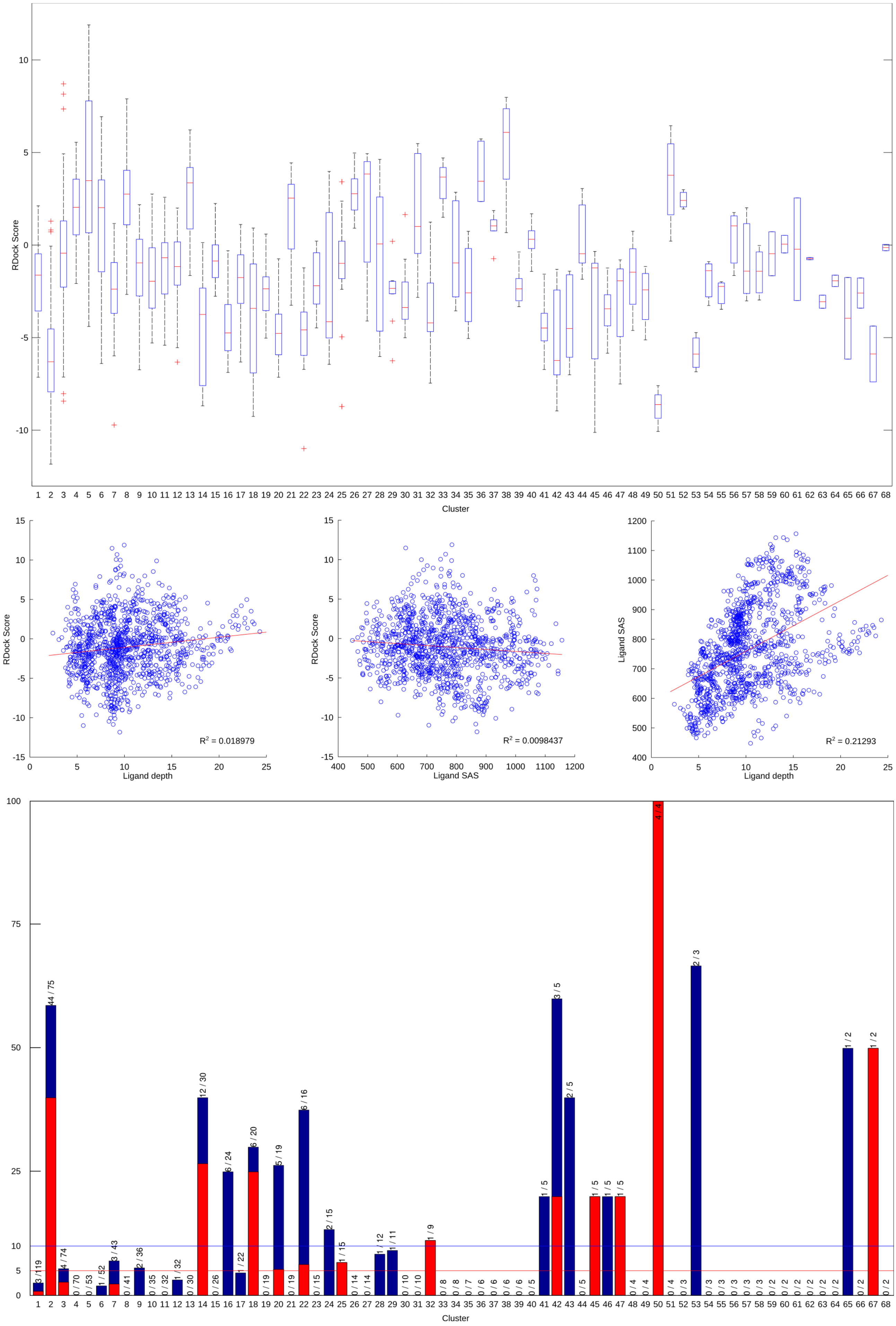

Additional figure 7.3: Docking result statistics for the CXCR4-based model.

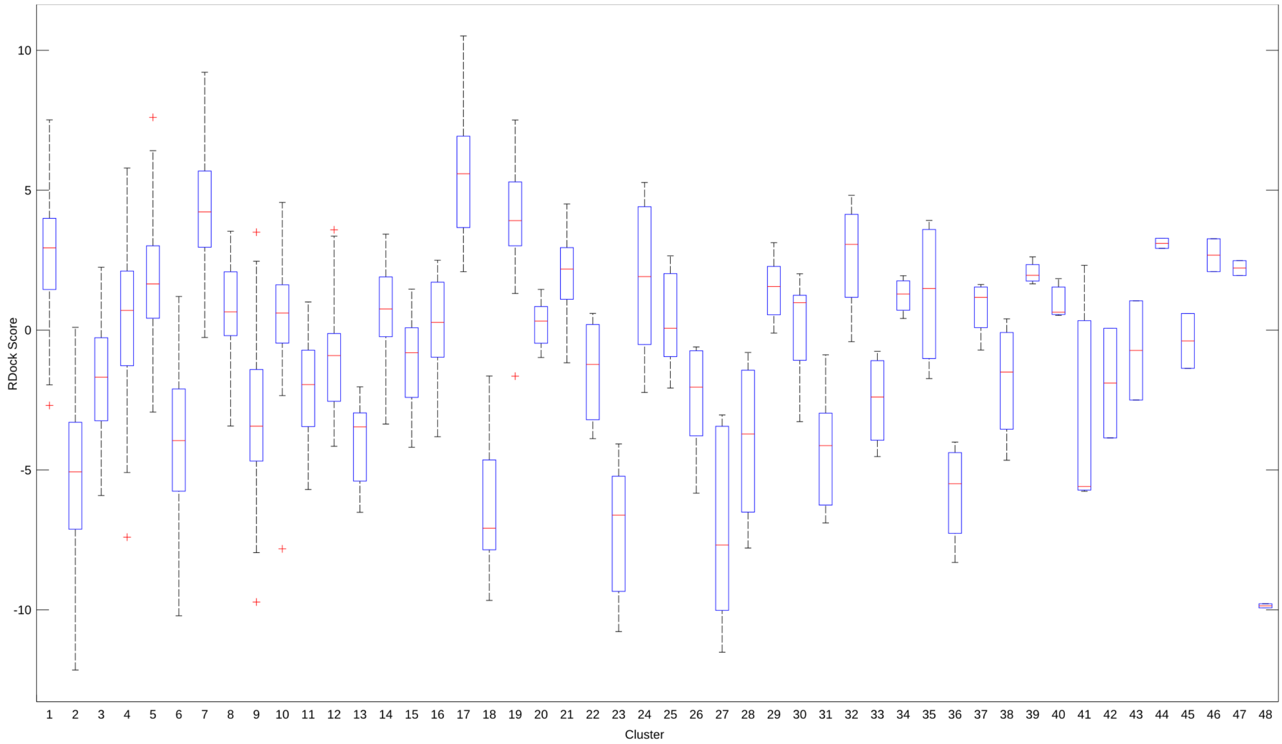

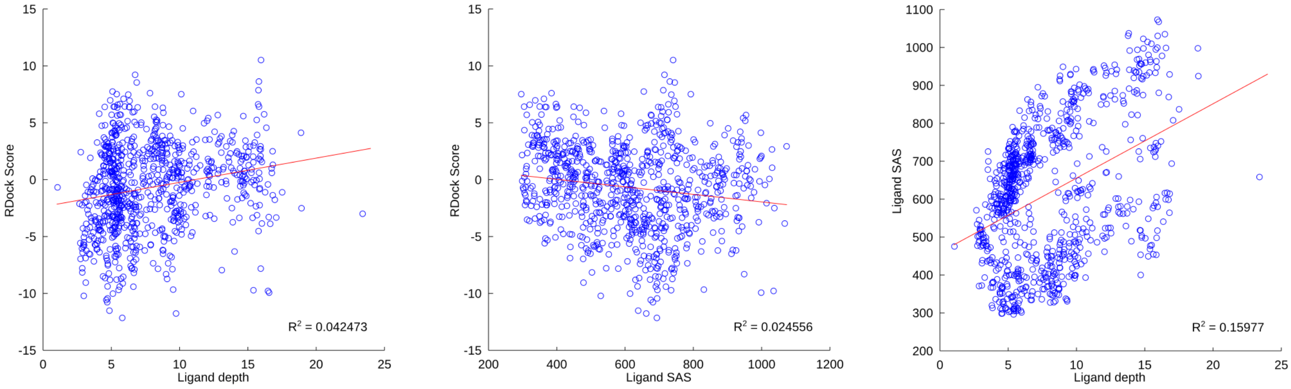

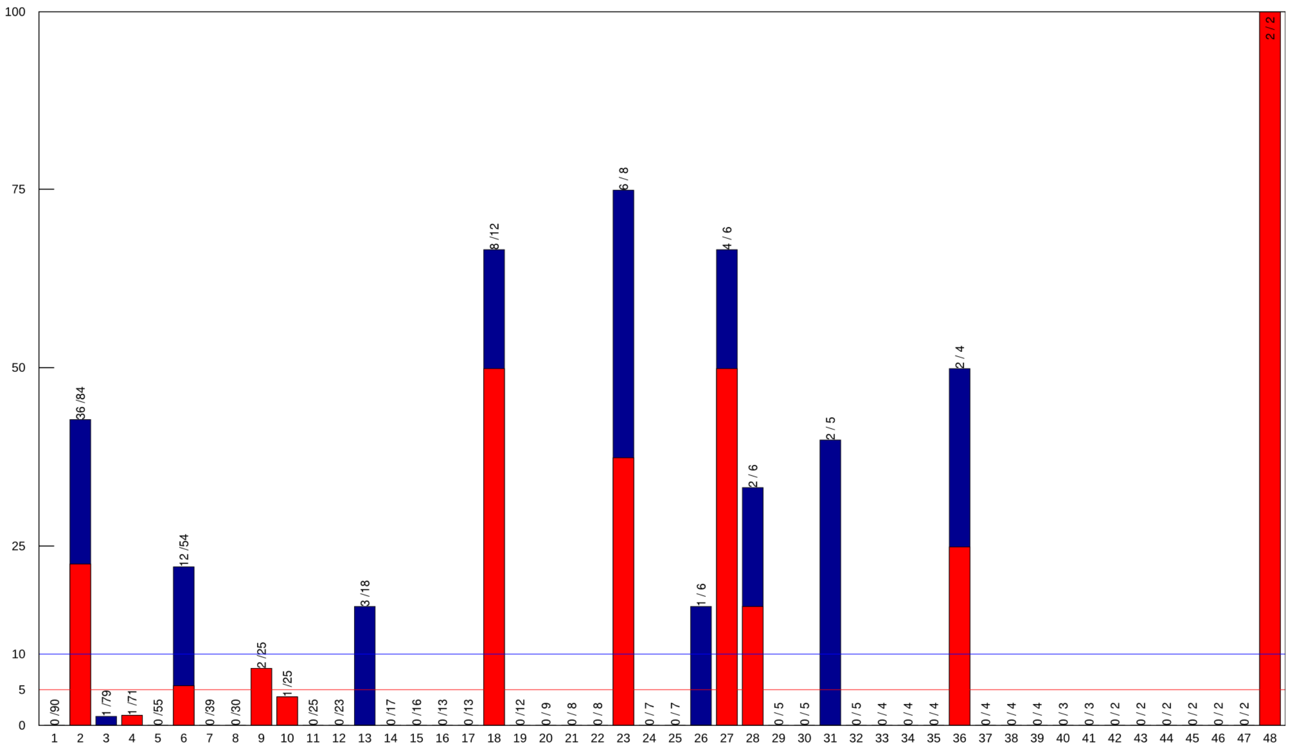

Additional figure 7.4: Docking result statistics for the NTSR1_TM6-based model.
