# Supplementary material for: Modeling of the OX1R–orexin-A complex suggests two alternative binding modes
Source: BMC Struct Biol. 2015 May 9;15:9. doi: 10.1186/s12900-015-0036-2 (PMC4469407; doi:10.1186/s12900-015-0036-2)
Supplement: Additional file 8: — Representative docking poses and interaction heatmaps, modelwise. Additional figures corresponding to Figures 8 and 9, but for all top-scoring docking poses for each model. Addition figure 8.1 shows the representative high-scoring pose for the OX2R-based model, AF8.2 for the NTSR1-based model, AF8.3 for the CXCR4-based model, and AF8.4 for the NTSR1_TM6-based model. [file 12900_2015_36_MOESM8_ESM.pdf]

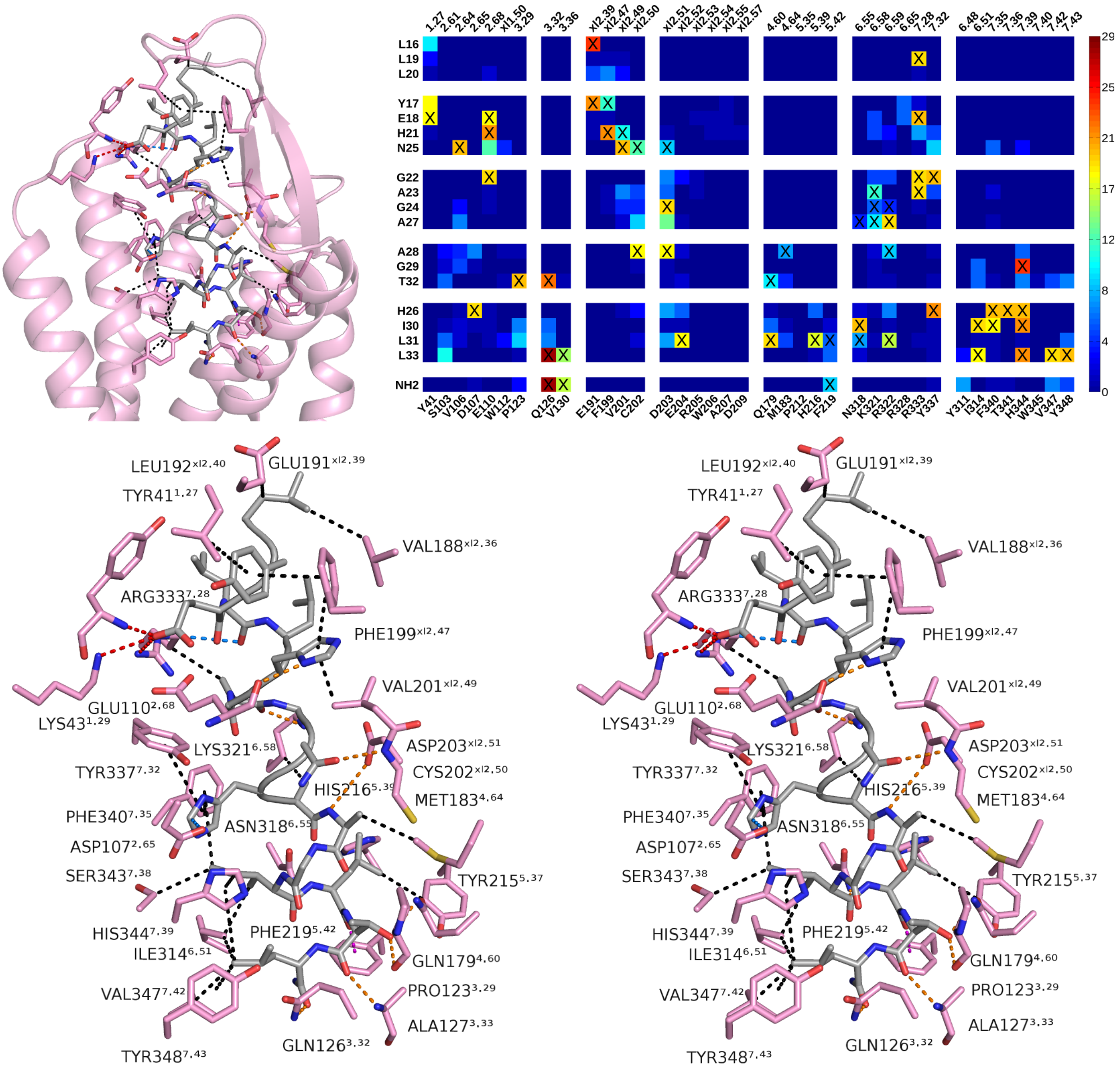

Additional file 8.1: Representative docking pose for the best ranking poses in the OX2R-based model. The binding mode here is the same as the TM7-mode discussed and illustrated in the paper (Figure 8), but the heatmap is drawn with top-ranking poses of the OX2R-based model.

(Top left) Overview of favorable receptor–ligand interactions. View from TM1-side of the receptor, TM1 hidden for clarity.
(Top right) Heatmap shows peptide–receptor contacts (interatomic distance < 4 Å) in the pool of high-ranking poses. X: observed in the representative pose.
(Bottom) cross-eyed stereogram. Orange: hydrogen bond, red: salt bridge / charge-assisted hydrogen bond, blue: CH–O hydrogen bond, magenta: lone pair-π, black: hydrophobic.

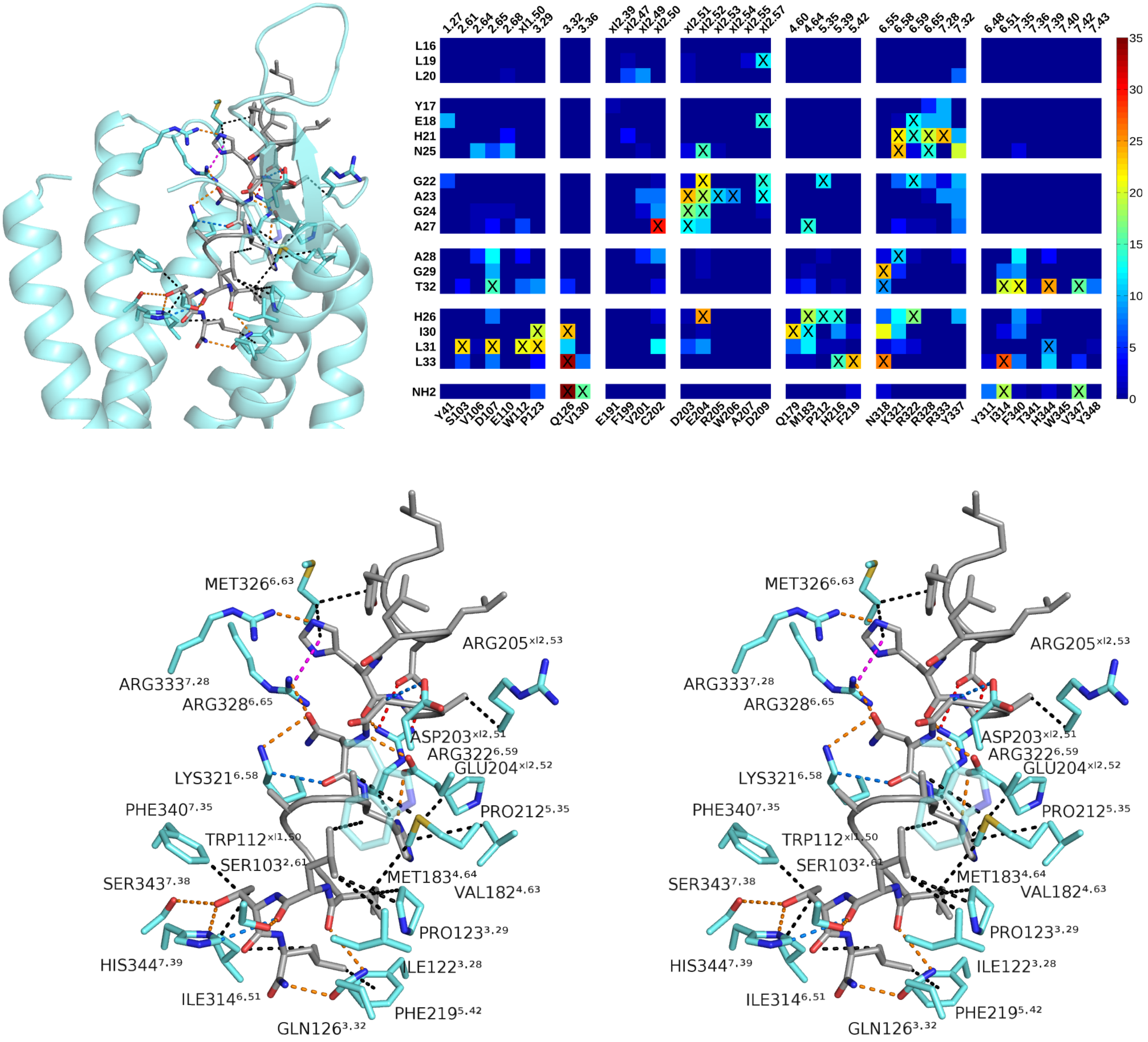

Additional file 8.2: Representative docking pose for the best ranking poses in the NTSR1-based model. The binding mode here is the same as the TM5-mode discussed and illustrated in the paper (Figure 9), but the heatmap is drawn with top-ranking poses of the NTSR1-based model.

(Top left) Overview of favorable receptor–ligand interactions. View from TM1-side of the receptor, TM1 hidden for clarity.
(Top right) Heatmap shows peptide–receptor contacts (interatomic distance < 4 Å) in the pool of high-ranking poses. X: observed in the representative pose.
(Bottom) cross-eyed stereogram. Orange: hydrogen bond, red: salt bridge / charge-assisted hydrogen bond, blue: CH–O hydrogen bond, magenta: cation-π, black: hydrophobic.

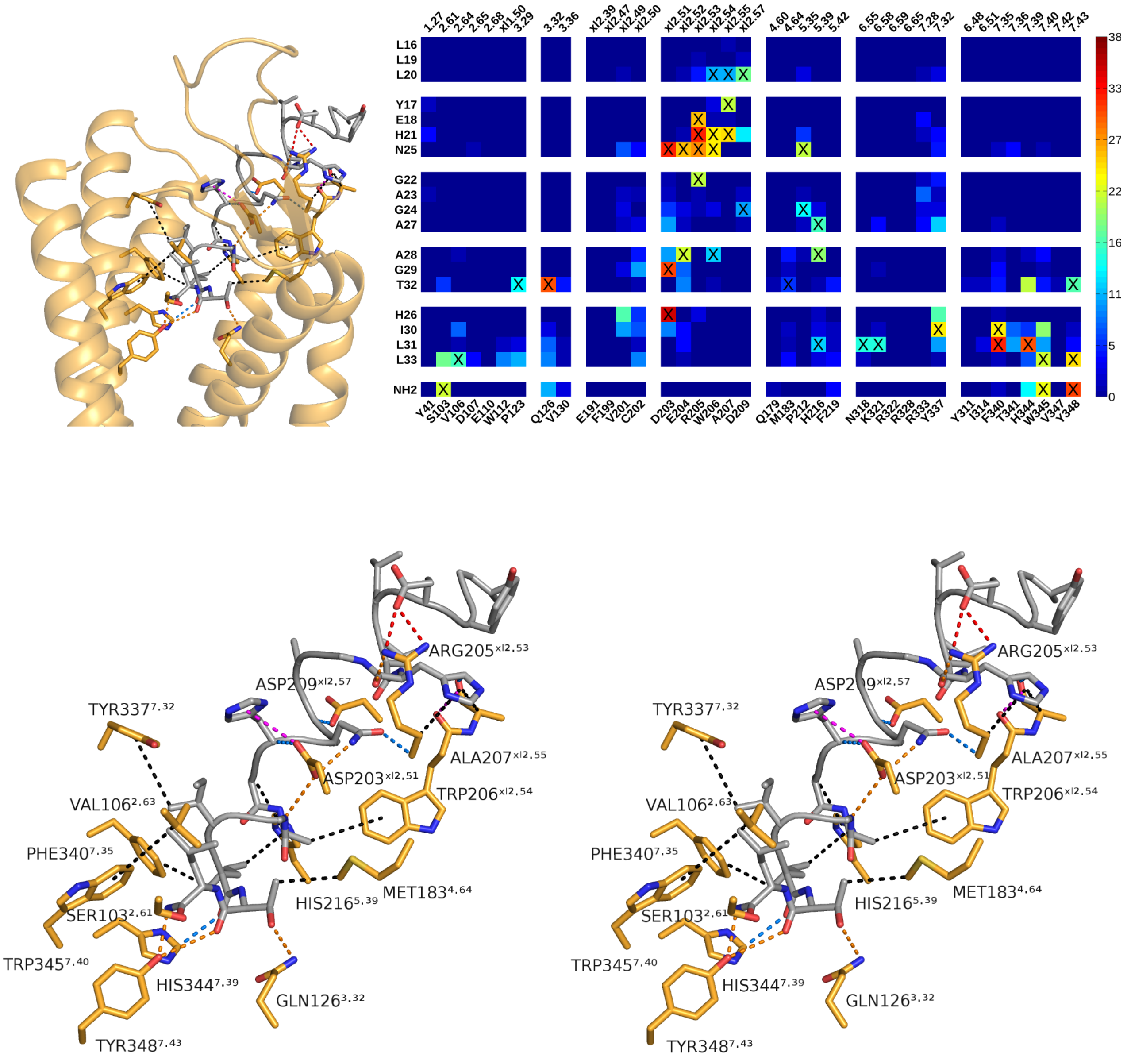

Additional file 8.3: Representative docking pose for the best ranking poses in the CXCR4-based model.

(Top left) Overview of favorable receptor–ligand interactions. View from TM1-side of the receptor, TM1 hidden for clarity.
(Top right) Heatmap shows peptide–receptor contacts (interatomic distance < 4 Å) in the pool of high-ranking poses. X: observed in the representative pose.
(Bottom) cross-eyed stereogram. Orange: hydrogen bond, red: salt bridge / charge-assisted hydrogen bond, blue: CH–O hydrogen bond, magenta: anion-π, black: hydrophobic.

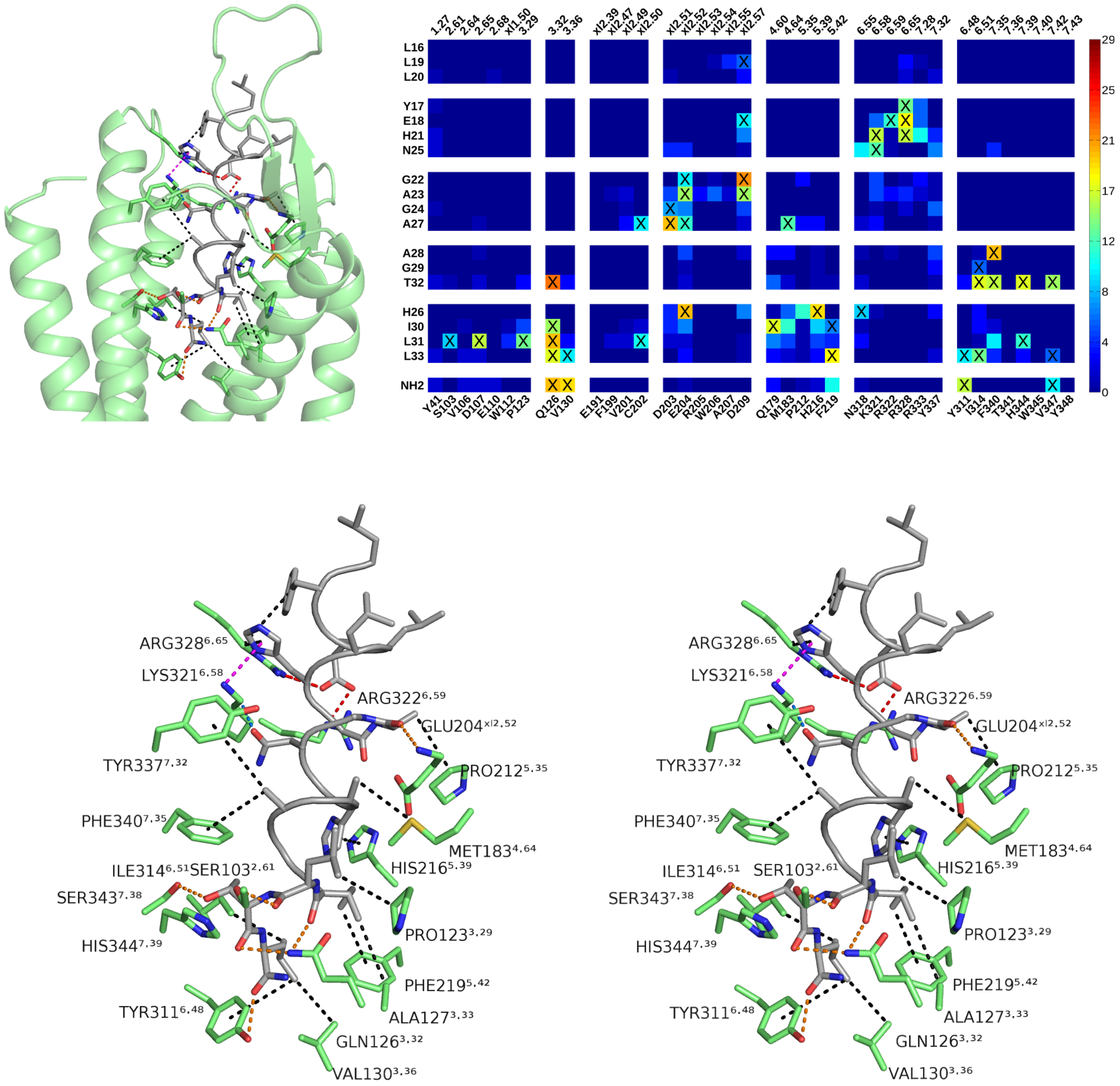

Additional file 8.4: Representative docking pose for the best ranking poses in the NTSR1_TM6-based model.

(Top left) Overview of favorable receptor–ligand interactions. View from TM1-side of the receptor, TM1 hidden for clarity.
(Top right) Heatmap shows peptide–receptor contacts (interatomic distance < 4 Å) in the pool of high-ranking poses. X: observed in the representative pose.
(Bottom) cross-eyed stereogram. Orange: hydrogen bond, red: salt bridge / charge-assisted hydrogen bond, blue: CH–O hydrogen bond, magenta: cation-π, black: hydrophobic.
